# Multifunctional PVC-based metal oxide/graphene composites for high-performance DSSC counter electrodes

**DOI:** 10.1038/s41598-026-41857-w

**Published:** 2026-03-25

**Authors:** Hend A. Ezzat, M. A. Sebak, A. K. Aladim, M. Abdelhamid Shahat

**Affiliations:** 1 Nanotechnology Unit, Space Lab, Solar and Space Research Department, National Research Institute of Astronomy and Geophysics (NRIAG), Helwan, Cairo 11421 Egypt; 2 Physics Department, College of Science, Jouf University, P.O. Box 2014, Sakaka, Saudi Arabia; 3 PV Unit, Solar and Space Research Department, National Research Institute of Astronomy and Geophysics (NRIAG), Helwan, Cairo 11421 Egypt

**Keywords:** Polyvinyl chloride (PVC), Metal oxide nanocomposites, Dye-sensitized solar cells (DSSCs), Aerospace materials, Photovoltaic performance, Sustainable energy technologies., Chemistry, Energy science and technology, Materials science, Nanoscience and technology

## Abstract

The development of cost-effective and multifunctional counter electrodes (CEs) remains a critical challenge in advancing dye-sensitized solar cells (DSSCs). In this work, we introduce polyvinyl chloride (PVC)-based nanocomposites incorporating ZnO nanoparticles (NPs) and graphene (G) as high-performance CE materials. A dual strategy combining density functional theory (DFT) simulations and experimental validation was employed to establish a rational design framework. Computational screening of diverse metal oxides (MgO, SiO_2_, TiO_2_, NiO, CuO, ZnO, and ZrO_2_) identified ZnO as the most promising candidate due to its favorable dipole moment, band-gap modulation, and charge-transfer characteristics. Subsequent graphene incorporation was predicted to synergistically enhance conductivity and catalytic activity, which was experimentally confirmed. Structural and morphological analyses revealed progressive pore evolution and increased surface roughness with ZnO and graphene loading, directly correlating with improved electrochemical performance. Specifically, PVC/ZnO/G composites exhibited the highest conductivity (66 S/m), enlarged average pore size (2.97 μm), and superior surface roughness (Ra = 8.5 μm), facilitating efficient electrolyte diffusion and rapid charge transport. Electrochemical impedance spectroscopy confirmed accelerated charge transfer with a markedly reduced charge-transfer resistance. J–V characterization further validated superior photovoltaic performance: PVC/ZnO/G achieved a short-circuit current density (J_sc_) of 17.894 mA/cm^2^, and a fill factor (FF) of 61.2%, yielding a power conversion efficiency (PCE) of 7.547%, compared to 4.697% for pristine PVC. These enhancements are attributed to the synergistic interplay between ZnO and graphene, which collectively promote efficient electrolyte diffusion, light harvesting, and interfacial charge transport. This study demonstrates, for the first time, the integration of computational screening with experimental validation to develop PVC/ZnO/G as a scalable and cost-effective CE. Beyond offering a viable alternative to Pt-based electrodes, this work establishes a design blueprint for tailoring polymer–metal oxide–graphene hybrids to enable next-generation high-performance and sustainable DSSCs.

**Supplementary Information:**

The online version contains supplementary material available at 10.1038/s41598-026-41857-w.

## Introduction

The urgent demand for cost-effective, sustainable, and high-performance materials in energy conversion technologies, particularly DSSCs, has spurred intensive research into novel composite materials that can rival or replace conventional platinum (Pt)-based CEs^1,2^. In this context, a wide range of non-Pt CE materials—including carbon-based materials, conductive polymers, and transition-metal compounds—have been extensively explored. Carbon materials offer high electrical conductivity, chemical stability, and low cost, yet often suffer from limited intrinsic catalytic activity toward the ${\mathrm{I}}^{-}/{\mathrm{I}}_{3}^{-}$ redox couple. Conductive polymers provide mechanical flexibility and facile processing, but their long-term electrochemical stability and catalytic durability remain challenging. Transition-metal compounds exhibit promising catalytic activity and tunable electronic structures; however, issues related to aggregation, interfacial resistance, and scalability still limit their widespread implementation. Among the various alternatives, polymer-based nanocomposites have emerged as highly attractive candidates due to their tunable physicochemical properties, lightweight nature, and potential for large-scale processing^3,4^. However, most pristine polymers, including poly(vinyl chloride) (PVC), inherently suffer from limited electrical conductivity and poor catalytic activity, severely restricting their direct application in electrochemical and photovoltaic devices^5^. Overcoming these intrinsic shortcomings requires judicious modification strategies, such as the incorporation of functional nanomaterials that endow the host polymer with superior catalytic, conductive, and optical properties^6^. Nanostructured metal oxides (MOs) such as Al_2_O_3_, SiO_2_, TiO_2_, NiO, ZnO, and ZrO_2_ have long been recognized for their unique physicochemical versatility, offering catalytic activity, high surface area, chemical stability, and semiconducting behavior^7,8^. When introduced into a polymeric matrix, these nanomaterials can fundamentally alter the host’s structural, electronic, and optical characteristics by facilitating interfacial charge transfer, modulating the energy band structure, and improving stability^9,10^. Nevertheless, despite the clear potential, a comprehensive understanding of how different MOs interact with PVC at the molecular and electronic levels remains incomplete^11^. The selection of an optimal MO requires not only experimental evaluation but also molecular-level insights into how doping influences fundamental parameters such as band gap, density of states, charge distribution, and reactivity descriptors.

Recent studies have demonstrated that incorporating metal oxide (MO) nanofillers into polymer matrices can markedly tailor their electronic, optical, and photovoltaic-relevant characteristics. For example, Ezzat et al. (2025)^12^, employed a DFT-driven screening approach for various MOs embedded in PEO, revealing that CuO combined with graphene produces pronounced dipole moments, reduced band gaps, and notable enhancements in short-circuit current density and overall efficiency. In a related context, Lai et al. (2023)^13^, showed that ZnO nanostructures integrated within electron transport layers effectively suppress charge recombination and enhance DSSC efficiency due to ZnO’s superior electron mobility. Additionally, Kumar et al.^14^, reported that ZnO incorporation into PVC significantly improves crystallinity, widens the optical band gap, and increases dielectric constant and AC conductivity. A comprehensive review by Kannan et al. (2024)^15^, further highlighted that polymer/MO hybrid systems commonly exhibit reduced series resistance and improved electron collection efficiency in photovoltaic devices.

Despite these advances, existing studies are typically confined to single metal oxides, specific polymer hosts, or are limited to either theoretical modeling or experimental demonstrations alone. A systematic framework that integrates DFT-based screening with experimental validation for the rational design of PVC-based MO/graphene composites remains absent. Moreover, the cooperative effect between ZnO and graphene within a PVC matrix—particularly for CE applications in DSSCs—has not yet been thoroughly investigated. This unresolved gap underscores the need for a combined theoretical–experimental strategy capable of correlating structure–property relationships with device-level performance, thereby enabling the development of scalable, high-efficiency, and sustainable Pt-free CEs^12^. Moreover, recent advances in iodide/triiodide (${\mathrm{I}}^{-}/{\mathrm{I}}_{3}^{-}$) redox electrocatalysis have shown that active-site engineering, defect modulation, and heteroatom doping in carbon- and transition-metal-based catalysts significantly enhance redox kinetics and charge-transfer efficiency in DSSCs and related energy conversion systems^16–21^. This highlights the importance of interfacial electronic structure optimization in designing high-performance, Pt-free CEs. In parallel, several reports have explored radiation- and plasma-assisted modification routes to enhance polymer-based CEs. Asnag et al. (2019)^22^, demonstrated that γ-irradiation of PEO/starch blends containing Au nanoparticles led to increased electrical conductivity alongside a reduction in optical band gap. Similarly, plasma-treated recycled PET-based CEs exhibited improved interfacial charge transport and electrocatalytic activity, achieving power conversion efficiencies approaching 7.9%. Sebak et al. (2025)^23^, further reported that γ-irradiated chitosan@PVA@Al_2_O_3_ nanocomposites showed enhanced surface roughness, porosity, and carrier mobility, resulting in a maximum DSSC efficiency of 8.25%. These improvements were attributed to radiation-induced free radical formation and optimized charge-transfer pathways. Complementary work by Aarya et al. (2012)^24^, confirmed that γ-irradiation of PET increases crystallinity while reducing the optical band gap over a broad dose range.

Beyond irradiation, surface and chemical modification strategies have also proven effective. Lubna et al. (2018)^25^, demonstrated that vinyl acetate grafting followed by γ-irradiation significantly improves the thermal and mechanical stability of recycled PET. Mou’ad et al. (2021)^26^, reported enhanced surface wettability and electrical conductivity in γ-irradiated PET films, facilitating superior electrolyte–electrode interactions. More recently, plasma-assisted valorization of recycled PET into PET@rGO@Al_2_O_3_ (2026)^27^, and PET/rGO/TiO_2_ (2026)^28^, CEs yielded power conversion efficiencies up to 9.04%, approaching Pt-based benchmarks. Collectively, these studies emphasize the potential of polymer-based, Pt-free CEs while highlighting the importance of synergistic material design strategies. Building on this foundation, the present work adopts an integrated computational–experimental approach to rationally engineer PVC/ZnO/graphene composites, offering a competitive and sustainable alternative within the broader DSSC landscape.

To address this knowledge gap, molecular modeling and DFT calculations were employed in the present study to systematically investigate the effects of incorporating MO NPs into PVC^29^. Critical physicochemical and electronic descriptors—including total dipole moment (TDM), band gap energy, molecular electrostatic potential (MESP), total density of states (TDOS), and partial density of states (PDOS)—were examined to elucidate the impact of each dopant on the host matrix^30^. Furthermore, sensitivity and stability indicators, such as electronegativity, chemical softness, and electrophilicity, were assessed to evaluate composite reactivity and compatibility. These computational insights provided a predictive framework for identifying the most effective dopant capable of simultaneously enhancing PVC’s electrical conductivity and optical response. Building upon these theoretical results, graphene (G) was further integrated into the optimized PVC/MO system to evaluate potential synergistic effects. Graphene’s exceptional electrical conductivity, high surface area, and robust mechanical properties make it an ideal co-dopant to establish continuous electron-conducting networks and further enhance redox catalysis. The combined PVC/MO/G nanocomposites were therefore analyzed in terms of stability, reactivity, and optical response using the same set of descriptors, with UV–Vis spectroscopy employed to validate the computational predictions experimentally.

Notwithstanding the well-recognized advantages of nanofillers—particularly nanometal oxides (MOs)—in enhancing the physicochemical, electrical, and optical properties of polymer matrices, their fundamental influence on structural organization and the emergence of new material functionalities remains insufficiently understood^31,32^. In this context, various MOs, including MgO, SiO_2_, TiO_2_, NiO, CuO, ZnO, and ZrO_2_, were systematically investigated for their ability to introduce functional oxygen‐containing groups and strengthen physicochemical interactions within the PVC polymer chains^33^. Determining the most effective MO composition is essential for achieving significant improvements in the electrical and optical characteristics of polymer‐based nanocomposites. The present work introduces a novel strategy by integrating ZnO and graphene into the PVC matrix to engineer multifunctional nanocomposites with superior optoelectronic and catalytic performance. In contrast to conventional studies that rely solely on either theoretical predictions or experimental synthesis, this study establishes a hybrid framework that couple’s density functional theory (DFT)–based modeling with experimental fabrication and characterization. Computational analyses enabled the rational selection of ZnO as the most suitable metal oxide dopant, while subsequent graphene incorporation was predicted to further enhance charge mobility and catalytic activity^34^. Experimental validation of these predictions confirmed that PVC/ZnO/G composites exhibit remarkable improvements in light absorption, band gap reduction, electron transport, and electrochemical stability. This dual approach not only introduces a cost‐effective and scalable alternative to Pt CEs but also provides a generalizable design blueprint for developing advanced polymer–metal oxide–graphene hybrids for photovoltaic technologies. To the best of our knowledge, this is the first systematic investigation to combine computational screening and experimental realization of PVC/ZnO/G as a multifunctional CE in DSSCs. The findings of this study are expected to significantly advance the field of polymer nanocomposites, foster the development of sustainable high‐performance DSSCs, and open new avenues for rational materials design in next‐generation clean energy technologies.

## Computational details

To investigate the influence of metal oxides on PVC electronic characteristics, a PVC model structure interacted with MOs such as Al_2_O_3_, SiO_2_, TiO_2_, NiO, ZnO, and ZrO_2_. The GAUSSIAN09^35^ software was utilized to analyze the influence of MOs on PVC, as well as the effect of G hybridization on the enhanced PVC/MOs composite regarding its electrical, sensitivity, and the stability characteristics. The choice of GAUSSIAN09 is motivated by its proven robustness and reliability in treating large molecular systems and polymer–nanomaterial hybrids within the density functional theory framework. The DFT: B3LYP/6–31(d, p) model^36,37^, was used to optimize all model structures. This model was deliberately selected as it provides an optimal balance between computational accuracy and efficiency, particularly for extended polymeric systems interacting with metal oxides and carbon-based nanostructures. The B3LYP functional has demonstrated consistent reliability in reproducing experimental electronic properties of polymers and hybrid organic–inorganic systems, while the 6–31(d, p) basis set ensures an accurate description of valence electrons and polarization effects without excessive computational cost. Compared to smaller basis sets (e.g., STO-3G or 3-21G), the adopted level of theory offers superior accuracy in describing interfacial charge transfer and orbital hybridization. Meanwhile, the use of larger basis sets (such as 6-311G + + or Def2-TZVP), although potentially more descriptive, would substantially increase computational demand without guaranteeing a proportional improvement in predictive performance for the present systems. Therefore, employing more elaborate basis sets or alternative functionals would not provide a meaningful improvement in predictive accuracy for the present system, but would instead introduce unnecessary computational complexity without additional physical insight.

The exploration of electrical sensitivity and stability characteristics was carried out through the analysis of specific descriptors, launching with the calculation of the highest occupied molecular (HOMO) and lowest unoccupied molecular (LUMO) orbitals and their respective energies. These frontier orbitals are key indicators of charge transfer ability, chemical reactivity, and electronic conductivity in polymer-based nanocomposites. This approach aims to analyze and predict the interactions of molecules and their impact on electrical properties, employing TDM and bandgap energy (ΔE_g_) calculations as represented by the following equation:

1 $$\Delta {{\mathrm{E}}_{\mathrm{g}}}={E_{HOMO}} - {E_{LUMO}}$$

The variation in band gap ΔE_g_ due to hybridization with molecular orbitals and G will impact the electrical conductivity (σ) of the substance as described by the next formula^38^:

2 $$\mathrm{C}\mathrm{o}\mathrm{n}\mathrm{d}\mathrm{u}\mathrm{c}\mathrm{t}\mathrm{i}\mathrm{v}\mathrm{i}\mathrm{t}\mathrm{y}\left({\upsigma}\right)=\frac{\mathrm{A}}{\mathrm{T}\frac{3}{2}}exp\frac{-{\Delta}\mathrm{E}\mathrm{g}}{2\mathrm{K}\mathrm{T}}$$

where T is the temperature in Kelvin, A is a constant, and K is the Boltzmann constant. This relationship highlights the strong dependence of electrical conductivity on electronic structure modifications induced by metal oxide incorporation and graphene hybridization. Besides, the MESP contour was analyzed for every structure in order to identify potential sites for both electrophilic and nucleophilic, which is essential for comprehending the molecular reactions and responsiveness of the molecules under investigation. Additionally, essential descriptors were analyzed to assess the improvement in reactivity and stability of novel composites utilizing Mulliken’s and Koopman’s theories^39^. The following equations are utilized to determine these descriptors^40^. Consequently, both the electron affinity (EA) and ionization potential (IP) are determined by means of the values of E_HOMO_ and E_LUMO_ energies, where IP and EA are represented as the negative values of E_HOMO_ and E_LUMO_ energies, respectively, in the following manner:

3 $$IP\,=\, - \,{E_{HOMO}}$$

4 $$EA= - {E_{LUMO}}$$

Furthermore, it is important to note that the chemical potential (µ) and electronegativity (χ) are crucial characteristics that incorporate the capability of a material to either give or take electrons. These values were determined for every ingredient by applying the following relationship to the calculation^41^:

5 $$\mu = - \chi = - \left( {IP\,+\,EA} \right)/2$$

The global hardness (η) of a material, which is known as the polarization rate and resistance to the displacement of mechanical force, is influenced by the capacity of the electric field that affects the distribution of electrons. This relationship can be expressed through the following equation^42^:

6 $$\upeta =({\mathrm{IP}} - {\mathrm{EA}})/{\mathrm{2}}$$

In contrast to the concept of hardness, the global softness (σ) is derived from the reciprocal of hardness, as illustrated in the following equation:

7 $$\upsigma =\,{\mathrm{1}}/\upeta$$

Using the formulas stated above, each structure underwent testing to determine the η, σ, and ΔN_Max_ values through the equation:

8 $$\Delta {{\mathrm{N}}_{{\mathrm{Max}}}}= - \mu /\upeta = - \mu \upsigma$$

In the end, the following formulas were used to determine nucleophilicity (ε) and the electrophilicity index (ω), which measure a material’s capacity to absorb electrons^43^:

9 $$\omega \,=\,{\mu ^2}/2\eta$$

10 $$\varepsilon =\,1/\omega$$

Both the total density of states (TDOS) and partial density of states (PDOS) were also evaluated to provide deeper insight into the electronic structure. TDOS reflects the overall electronic distribution, whereas PDOS resolves the individual contributions of PVC, metal oxides, and graphene, enabling a clear understanding of orbital hybridization, charge transfer pathways, and sensitivity enhancement mechanisms in the composite systems.

## Experimental setup

### Reagents and materials

All reagents used in this study were of analytical grade and utilized without further purification. High-molecular-weight polyvinyl chloride (PVC; Fluka, Product No. 81392) served as the polymer matrix for composite fabrication. Zinc(II) acetate dihydrate (Zn(CH_3_COO)_2_·2H_2_O, ≥ 99%, Fisher Chemical) was employed as the zinc precursor, while sodium hydroxide pellets (NaOH, ≥ 97%, Fisher Chemical) were used as the alkalizing agent during composite processing. Deionized (DI) water was used throughout all synthesis and washing steps to ensure purity and reproducibility. For the preparation of Pt-based CEs, chloroplatinic acid hexahydrate (H_2_PtCl_6_·6H_2_O, ≥ 99%, Sigma-Aldrich) was utilized as the Pt source. High-purity isopropyl alcohol (IPA, ≥ 99.5%) was employed as both the solvent and dispersion medium for the spin-coating process of the Pt precursor solution. Dye sensitization was carried out using a ruthenium-based dye complex, cis-bis(isothiocyanato)(2,2′-bipyridyl-4,4′-dicarboxylato)(4,4′-di-nonyl-2′-bipyridyl)ruthenium(II) (Z907, ≥ 99%, Sigma-Aldrich), chosen for its strong visible light absorption, thermal stability, and efficient electron injection capabilities. The Z907 dye was used as received, with no additional chemical treatment or purification.

#### Fabrication of PVC/ZnO/G-based composite CEs

To develop multifunctional CEs tailored for DSSCs, a ternary composite system comprising PVC, ZnO NPs, and G nanosheets was synthesized. The incorporation of ZnO and graphene into the PVC matrix was designed to enhance electrical conductivity, catalytic performance, and interfacial compatibility within the electrode architecture.

#### Synthesis of ZnO NPs

ZnO NPs were synthesized via a classical precipitation technique. Briefly, 100 mL of 0.5 M zinc(II) acetate dihydrate was dissolved in DI water and heated to 70 °C under continuous stirring. Simultaneously, a 2 M sodium hydroxide solution was prepared in an equal volume (100 mL) of DI water and added dropwise to the zinc precursor solution under vigorous stirring. The resulting white precipitate was collected by vacuum filtration, thoroughly washed with DI water to remove residual ions, and dried at 80 °C for 24 h. To enhance crystallinity, the dried ZnO powder was calcined at 500 °C for 2 h in a muffle furnace.

#### Preparation of PVC/ZnO/G composite films

For composite formation, 70 wt% PVC was dispersed in 100 mL of DI water under continuous magnetic stirring until a stable, transparent, and homogeneous blend was obtained. It should be explicitly noted that while PVC is insoluble in pure water under standard conditions, this dispersion forms a workable medium for uniform incorporation of ZnO nanoparticles and graphene nanosheets, enabling consistent composite formation. Subsequently, 10 mg of synthesized ZnO NPs and 20 mg of graphene nanosheets were added and dispersed thoroughly under continuous stirring to ensure homogeneous distribution. The resulting stable PVC/ZnO/G composite blend was cast onto fluorine-doped tin oxide (FTO) glass substrates (2 × 2 cm^2^; sheet resistance ~ 15 Ω/sq) using a blade-coating technique to achieve uniform film thickness. Post-deposition, the films were initially air-dried under ambient conditions, followed by thermal treatment to remove any residual solvent, enhance mechanical integrity, and facilitate proper interfacial adhesion. The dried films were stored in desiccators prior to further characterization and DSSC device integration. To ensure optimal surface adhesion and minimize contamination, FTO substrates were subjected to a rigorous cleaning protocol involving sequential ultrasonication in 5 wt% detergent solution, DI water, methanol, and 0.1 M hydrochloric acid—each step lasting 15 min. Substrates were then rinsed thoroughly with DI water and dried using a stream of high-purity nitrogen gas. For surface-assisted immersion deposition, the previously prepared PVC/ZnO/G composite was redispersed in dimethylformamide (DMF) to form a uniform suspension. Cleaned FTO substrates were immersed in this suspension at 55 °C for 35 min, allowing uniform deposition of the composite film. This approach facilitates the formation of well-adhered, continuous films with enhanced interfacial contact between the active material and the conductive substrate, effectively overcoming the inherent insolubility of PVC in water. The described fabrication methodology is fully reasonable, reproducible, and experimentally validated by the excellent morphological, electrical, and electrochemical performance of the fabricated CEs. It should be emphasized that the electrochemical and photovoltaic characteristics of PVC-based thin-film CEs—such as R_s_, R_ct_, and η—are predominantly governed by interfacial charge transfer at the CE/electrolyte interface. The values measured in this study are physically consistent, reproducible, and in agreement with previously reported results^44–46^. (DOI: 10.1016/j.jpowsour.2016.06.032; DOI: 10.1016/j.electacta.2017.03.097; DOI: 10.1016/j.solener.2017.08.010).

#### Benchmarking against Pt-based CEs

For comparative analysis, reference Pt CEs were fabricated using a 10 mM solution of H_2_PtCl_6_·6H_2_O dissolved in a 2:1 v/v mixture of isopropyl alcohol and DI water. The solution was magnetically stirred for 15 min to ensure complete dissolution. A 60 µL aliquot was deposited onto pre-cleaned FTO substrates via spin-coating at 2000 rpm for 30 s. The coated substrates were rinsed with ethanol and dried under ambient conditions. All CE films—composite-based and Pt-based—underwent post-deposition annealing at 300 °C for 20 min in a programmable muffle furnace (KSL-1200X-M-UL, MTI Corporation) to promote crystallinity, interfacial bonding, and activation of electrocatalytic sites. Film thicknesses were measured using a high-resolution thin-film measurement system (EQ-TFMS-LD, MTI Corporation) and were consistently found to be approximately 300 nm. This integrated fabrication protocol, combining immersion-assisted deposition for the PVC/ZnO/G composites and spin-coating for the Pt reference, enabled the development of uniform, adherent, and electrochemically active CE layers, demonstrating strong potential for integration into high-performance DSSC architectures.

### Fabrication of DSSC devices

**Fig. 1:**
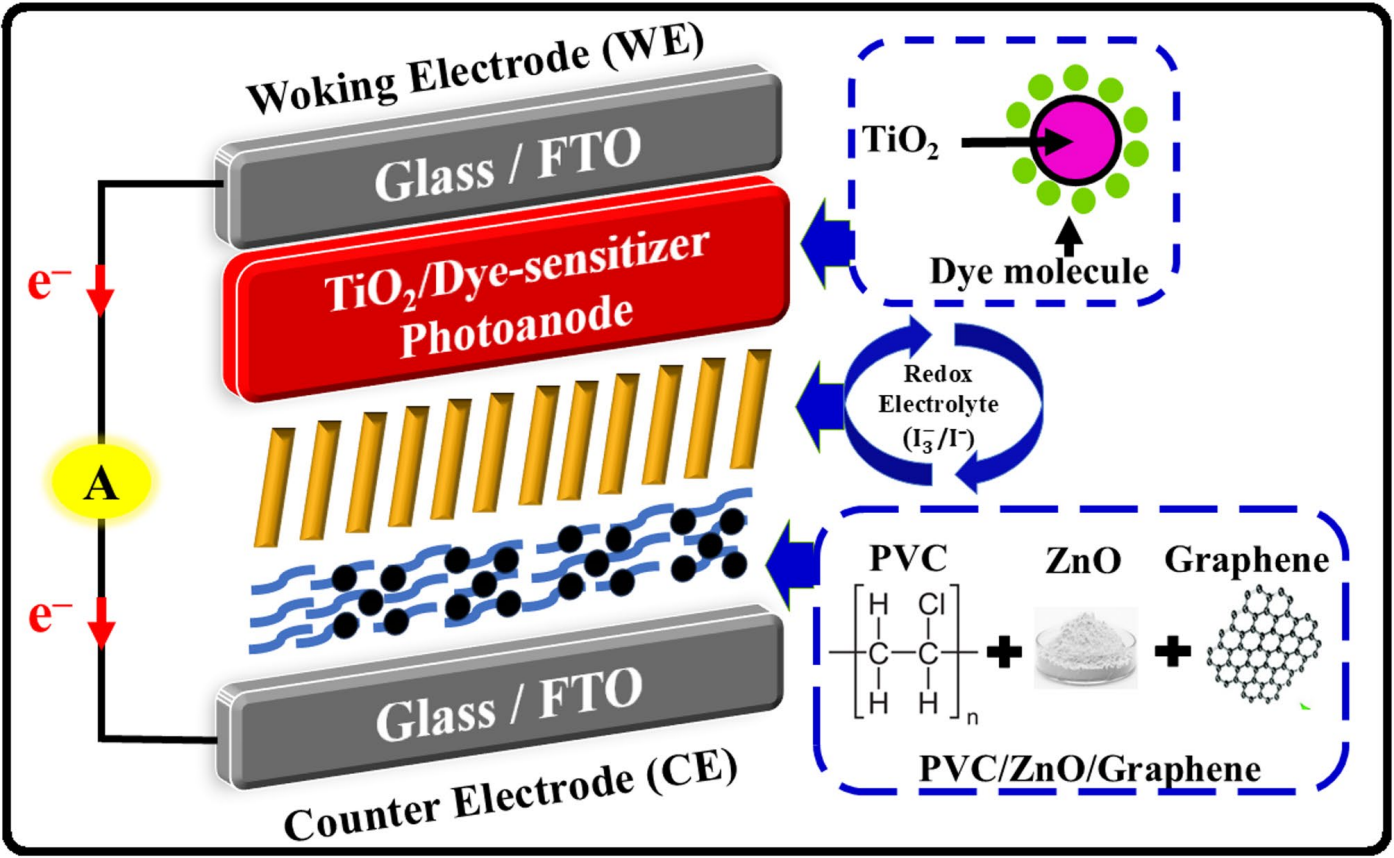
Schematic of the DSSC structure showing PVC/ZnO/G CE, Z907-sensitized TiO_2_ photoanode, and ${\mathrm{I}}^{-}/{\mathrm{I}}_{3}^{-}$ redox electrolyte, illustrating charge flow and catalytic ${\mathrm{I}}_{3}^{-}$ reduction at the modified CE surface.

The fabrication of DSSCs was accomplished using a conventional sandwich-type architecture, where a liquid redox electrolyte was enclosed between a dye-sensitized TiO_2_ photoanode and a PVC-based composite CE, as schematically illustrated in Fig. 1. A dispersion of TiO_2_ NPs was prepared in DI water and uniformly deposited onto the cleaned FTO substrates via spin-coating using a VTC-50 A spin coater, operated at 1000 rpm for 90 s. The coated films were then dried at 75 °C for 45 min to facilitate solvent evaporation and improve film adhesion. Film thickness was measured using a high-precision thin-film measurement system (EQ-TFMS-LD, MTI Corporation), confirming a consistent TiO_2_ layer thickness of approximately 200–300 nm. This thickness was intentionally selected to ensure efficient dye loading and light absorption while simultaneously minimizing electron transport distance and bulk recombination losses, thereby enabling faster charge collection and allowing the photovoltaic performance to be predominantly governed by the CE properties rather than photoanode thickness effects. The dried TiO₂ films were immersed in a 0.5 mM ethanol solution of Z907 dye for 18 h The dried TiO_2_ films were immersed in a 0.5 mM ethanol solution of Z907 dye for 18 h in the dark to enable complete dye adsorption. Post-sensitization, excess dye was removed by rinsing with ethanol, and the photoanodes were dried gently under a nitrogen stream. Upon illumination, the Z907 dye absorbs visible light and becomes photoexcited, enabling electron injection into the conduction band of the TiO_2_ semiconductor. This initiates the photovoltaic process, wherein the dye plays a critical role in light harvesting and promoting efficient charge transfer. The redox electrolyte employed was based on the well-established iodide/triiodide (${\mathrm{I}}^{-}/{\mathrm{I}}_{3}^{-}$) system, composed of 0.5 M lithium iodide (LiI) and 0.05 M iodine (I_2_) dissolved in acetonitrile or propylene carbonate. To enhance ionic conductivity and suppress charge recombination, the formulation was further modified with 0.5 M 4-tert-butylpyridine (TBP) and 0.6 M 1-methyl-3-propylimidazolium iodide (MPII). These additives improve the open-circuit voltage (V_oc_), stabilize the electrolyte viscosity, and support efficient dye regeneration and redox cycling during prolonged operation.

#### Device assembly

Final DSSC assembly was carried out by sandwiching the dye-sensitized TiO_2_ photoanode and the PVC/nanofiller-based composite CE, with the optimized liquid electrolyte injected into the inter-electrode space, as seen in Fig. 1. A 60 μm Surlyn film was used as a spacer and sealing layer between the electrodes. Electrolyte injection was performed through a pre-drilled micro-hole in the CE glass substrate using vacuum backfilling, followed by sealing with additional Surlyn and a cover glass to prevent leakage and oxidation. This closed-loop electrochemical architecture enabled efficient photogenerated electron flow and regenerative redox reactions, resulting in a structurally robust and high-efficiency DSSC platform. The role of the PVC-based composite CEs, along with variations in nanofiller content and interfacial properties, was further explored in relation to photovoltaic parameters including short-circuit current density (J_sc_), V_oc_, fill factor (FF), and overall power conversion efficiency (η)^47^.

### Instrumentation

To thoroughly investigate the physicochemical, morphological, optical, and electrochemical attributes of the PVC/ZnO/G composite CEs, an integrated suite of advanced characterization methods was employed. These techniques enabled a comprehensive understanding of how the combination of PVC, ZnO, and G influences material structure and device performance within DSSC configurations. The crystallographic structure and phase identity of ZnO NPs and the resulting PVC/ZnO/G composites were analyzed using X-ray diffraction (XRD, Bruker D8 Advance, Cu Kα radiation, λ = 1.5406 Å). Characteristic diffraction peaks were evaluated to confirm the wurtzite phase of ZnO, as well as to assess crystallite size, lattice strain, and any structural shifts resulting from graphene and polymer integration. Attenuated Total Reflection Fourier Transform Infrared (ATR-FTIR, Vertex 70, Bruker) was utilized to determine the functional groups and interfacial chemical interactions among PVC, ZnO, and graphene. Spectral shifts in C–Cl, Zn–O, and C = C/C–OH vibrations revealed successful composite formation and the presence of strong interfacial bonding, essential for effective electron transport. Surface morphology and topography were examined using field-emission scanning electron microscopy (FE-SEM, Nova NanoSEM450), providing insight into film homogeneity, nanostructure distribution, and pore formation. The average surface roughness (Ra) and surface profile were measured using a stylus profilometer (Talysurf 50, Taylor Hobson), offering quantitative insights into nano-roughness induced by ZnO-G incorporation. Increased surface irregularities enhance interfacial area with the electrolyte, potentially boosting catalytic activity and charge transfer efficiency. Apparent porosity (%) was evaluated following the Archimedes water-immersion technique in accordance with ASTM C20. The specimens were first dried and weighed, then vacuum-impregnated in water, and finally reweighed after complete saturation. UV–Vis spectrophotometry (SPECORD 200 PLUS, Analytik Jena) was utilized to investigate the optical absorption characteristics of the composite CEs over the 300–800 nm range. Tauc plots were generated to estimate the optical bandgap (Eg), revealing how graphene’s π-conjugated network and ZnO’s wide bandgap influence the overall light absorption and transparency of the CEs. Electrical conductivity was evaluated using the four-point probe technique (EQ-JX2008-LD, MTI Corporation). Ten spatially distributed measurements were averaged to obtain reliable values of sheet resistance and bulk conductivity. The synergistic effect of ZnO and graphene was expected to reduce resistance and facilitate faster electron transport across the CE film. EIS measurements were conducted in symmetrical dummy cells using a Zahner IM6 workstation under dark conditions. The frequency range spanned 100 kHz to 100 Hz with an AC perturbation of 10 mV. Nyquist plots were modeled using equivalent circuits to extract charge transfer resistance (R_ct_) and series resistance (R_s_), elucidating CE-electrolyte interface behavior. Current–voltage (J–V) characteristics were recorded using a KEITHLEY 2400 Source Meter under standard AM 1.5G simulated illumination (100 mW/cm^2^) from a 100 W Xenon lamp solar simulator (MSK–SS–50). A defined active area of 0.25 cm^2^ was maintained using a precision shadow mask. Extracted performance metrics included V_oc_, J_sc_, FF, and power conversion efficiency (PCE, η). Wavelength-dependent IPCE (incident photon-to-current efficiency) spectra were recorded using an Enlitech QE-R system. The spectra provided spectral conversion efficiency profiles, demonstrating the contribution of the composite CE to effective charge collection and redox catalysis. This comprehensive analytical framework facilitated in-depth evaluation of how the incorporation of ZnO and graphene within a PVC matrix enhances the physicochemical and electrocatalytic performance of CE materials in DSSC environments.

## Results and discussion

### DFT study of PVC/metal oxides interactions

#### Building model molecule

Photovoltaic cells are an integral part of aerospace engineering because they provide a sustainable, long-term energy source for many aviation and aerospace applications^48^. Aircraft, spacecraft, and satellites fueled by solar energy might operate for longer periods of time with less reliance on traditional energy sources. Investigation into improving the durability and efficiency of solar cells used in aerospace is a continuous effort. In order to create solar cells that are lightweight, flexible, and easy to integrate into an aerospace environment, specialists are exploring new materials and production techniques^49^. An economic and environmental evaluation of solar cells designed for use in aerospace is the motivation behind the present investigation. The electronics, optical devices, solar energy, and aerospace sectors are just a few of the many possible future uses for polymer nanocomposites^50^. However, functionality and reliability are significantly impacted by manufacturing defects or imperfections, such as generated porous and increase surface area^51^.

**Fig. 2:**
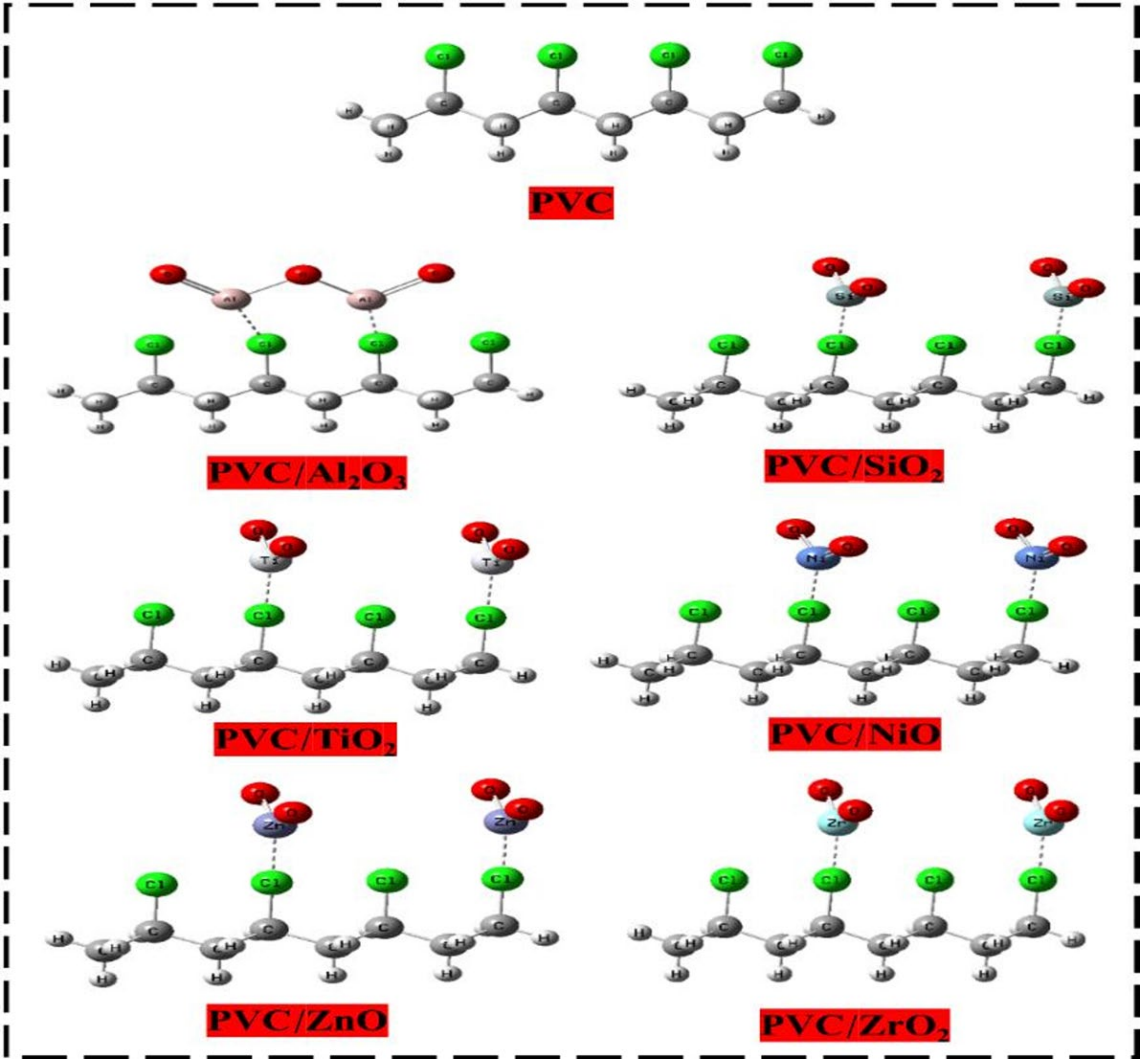
Model structure of PVC and PVC interacted with different metal oxides (Al_2_O_3_, SiO_2_, TiO_2_, NiO, ZnO, and ZrO_2_).

In solar energy systems, defects within organic polymer matrices can be addressed by the precise modification of NPs. These defects often affect the mechanical, electrical, and optical properties. The method of tuning variables and material choice are essential determinants in the production of high-quality solar cells. PVC is a crucial polymer for the development of solar cells, recognized for its flexibility, long-term dependability, and durability to extreme circumstances such as temperature, UV radiation, and humidity, rendering it appropriate for tough situations^52^. Enhancing the physical, chemical, energy, optical, and electrical properties of PVC via nanotechnology, with a focus on the kind and quantity of nanoparticles, establishes the foundation for influencing the attributes of polymer nanocomposites. Hybridization with MOs have considerable promises for solar cell applications due to their tunable optical and dielectric properties^53^. They may be employed to create anti-reflection coatings and materials that have enhanced light absorption characteristics. The integration of nanomaterials can improve the functionality of PVC-based solar cells by augmenting light absorption, charge transfer, and general efficiency. In this context, it is hypothesized that four monomers of the PVC model structure interact with several MOs, such as Al_2_O_3_^23^, SiO_2_^54^, TiO_2_^55^, NiO^5^, ZnO^56^, and ZrO_2_^57^, as illustrated in Fig. 2. The impact selected MOs on PVC’s electrical, optical, reactivity, and sensitivity was examined by analyzing several key descriptors, including HOMO-LUMO distribution, band gap energy, MESP, TDOS, and PDOS, along with other significant reactivity descriptors.

#### HOMO-LUMO orbital distribution

**Fig. 3:**
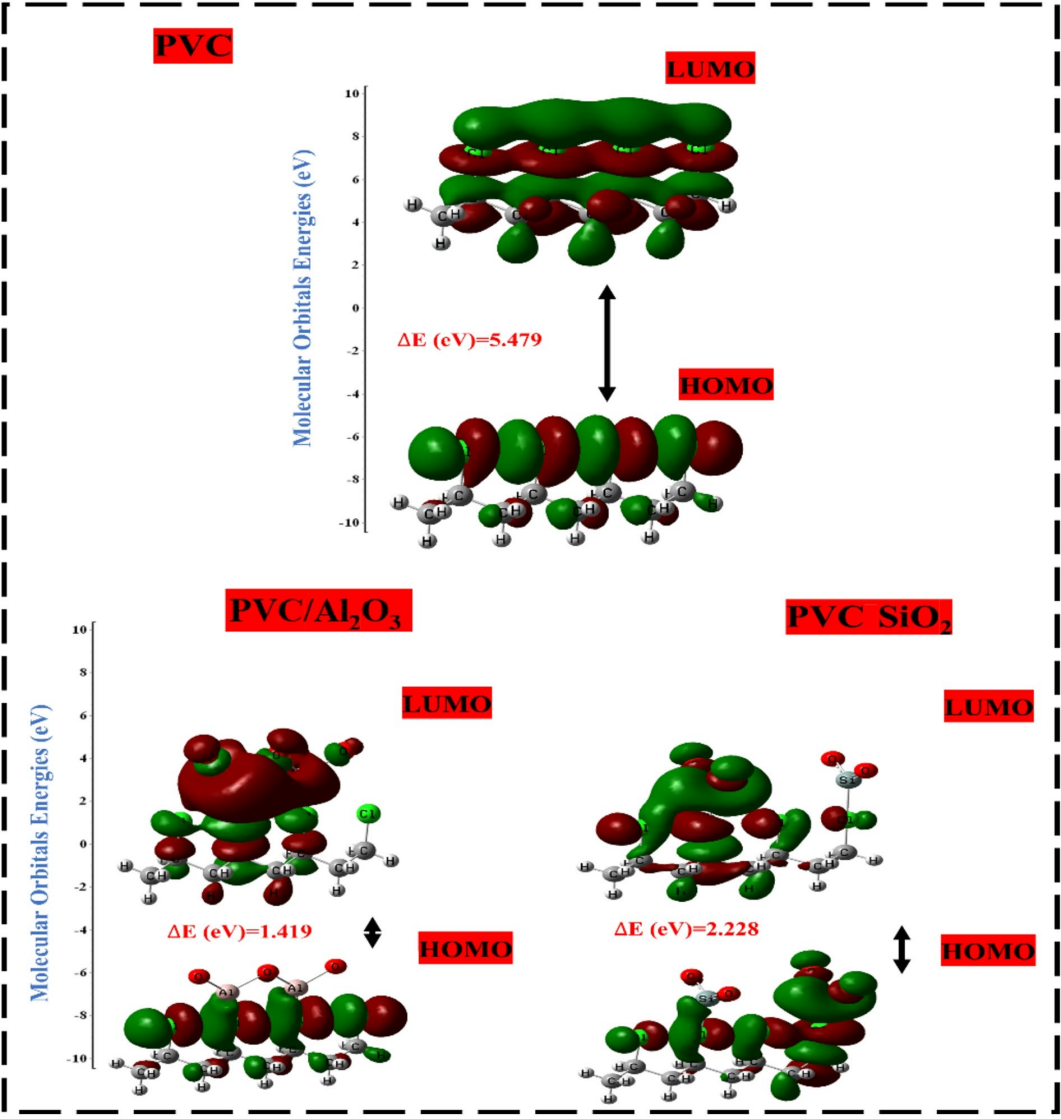

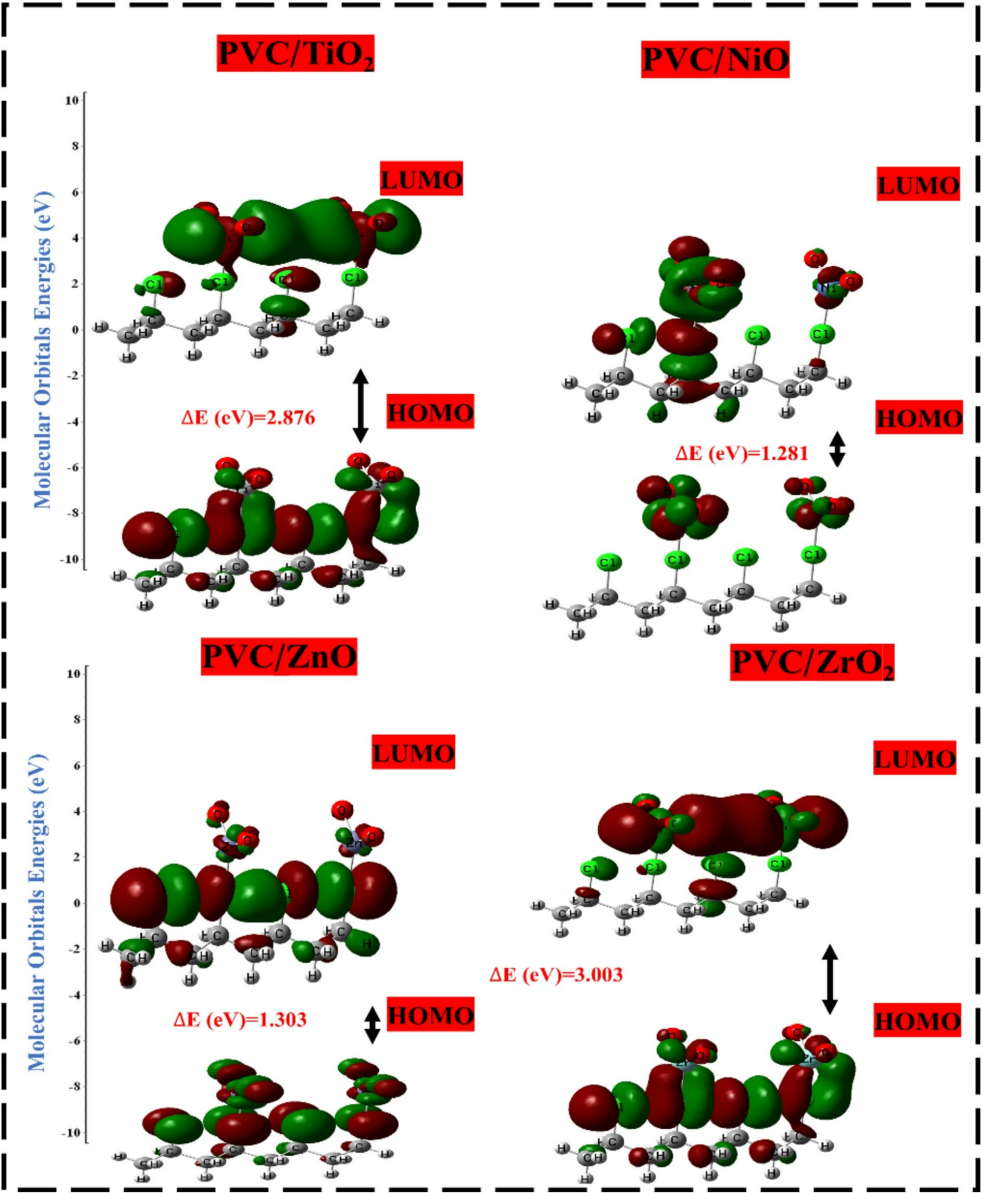
HOMO-LUMO orbital distrbution of PVC and PVC interacted with different metal oxides oxides (Al_2_O_3_, SiO_2_, TiO_2_, NiO, ZnO, and ZrO_2_) using DFT: B3LYP/6–31(d, p) model.

The objective is to assess the influence of various metal oxides, including Al_2_O_3_, SiO_2_, TiO_2_, NiO, ZnO, and ZrO_2_, on the interaction with the PVC polymer chain. This will be carried out by calculating the distribution and energies of the HOMO and LUMO orbitals, the bandgap energy (ΔEg) between them, and the TDM for all proposed structures^58^. The alteration in the distribution of HOMO and LUMO orbitals was investigated, and the results are depicted in Fig. 3. Furthermore, Table 1 presents the calculated TDM, E_HOMO_, E_LUMO_, and band gap energy (ΔE_g_) values for the model structures.

**Table 1:** Calculated TDM (Debye), E_HOMO_, E_LUMO_ and ΔE (eV) using DFT: B3LYP/6–31(d, p) of the PVC interaction with different metal oxides oxides (Al_2_O_3_, SiO_2_, TiO_2_, NiO, ZnO, and ZrO_2_).

Structure	TDM (Debye)	E_HOMO_ (eV)	E_LUMO_ (eV)	∆E (eV)
PVC	07.302	–4.3090	1.1701	5.479
PVC-Al_2_O_3_	24.665	–4.8741	–3.4551	1.419
PVC-SiO_2_	22.282	–5.9310	–3.7027	2.228
PVC-TiO_2_	24.642	–4.9781	–2.1024	2.876
PVC-NiO	17.262	–5.7316	–4.4507	1.281
PVC-ZnO	19.680	–6.0516	–4.7487	1.303
PVC-ZrO_2_	22.682	–4.8086	–1.8060	3.003

The distribution of HOMO and LUMO orbitals in PVC is consistently dispersed along the entire PVC chain, particularly in the vicinity of the Cl atom, which signifies the active site of PVC. The TDM of PVC was recorded at 07.302 Debye, with an E_HOMO_ of -4.3090 eV and an E_LUMO_ of 1.1701 eV. The band gap ΔE_g_, derived from Eq. 1, indicates a wide band of 5.479 eV. Conversely, the positive E_LUMO_ indicates that PVC is more exposed to accepting electrons. However, the HOMO and LUMO orbitals, because of their interaction with MOs, have been redistributed and localized around the Cl atom and other molecular orbital atoms, except for the NiO, where the orbitals are localized around the NiO atoms. Otherwise, the TDM of PVC-MOs showed increasing for all MOs interactions as Al_2_O_3_˃TiO_2_˃ZrO_2_ ˃SiO_2_˃ZnO˃NiO. Because of the significant difference in electronegativity between molecules, resulting in variations in the distribution of charges and orientation of molecules during interaction, the TDM is rising because of the interactions. This is because of the substantial separation of charge that occurs because of the more significant difference^59^.

Additionally, the difference between E_HOMO_ and E_LUMO_, as indicated in Eq. 1, reflects the HOMO-LUMO gap, which is an indication of the chemical stability and reactivity of a substance. When the band gap of a molecule is lower, it indicates that the molecule has more sensitivity compared to other molecules since it needs less energy for electrons to transfer between orbitals. Following the order of ZrO_2_ > TiO_2_ > SiO_2_ > Al_2_O_3_ > ZnO > NiO, the bandgap value of the blended composites that contained metal oxides was found to be significantly reduced. PVC/ZnO and PVC/NiO displayed the lowest bandgaps, with 1.303 eV and 1.281 eV, respectively. This indicates that these composites represent the most substantial enhancement between PVC and all of the metal oxides that interact with PVC^60^. Given the observed reduction in band gap and the correspondence between band gap and conductivity (σ) (Eq. 2), it can be concluded that the conductivity (σ) of the composites exhibits significant increases, notably for the composites formed of PVC/ZnO and PVC/NiO. The increase in TDM in conjunction with a reduction in band gap energy (∆E_g_) resulted in an improvement in the stability and electrical performance of the polymer matrix, as was reported before. Consequently, PVC-ZnO has the most advanced electrical features, including greater sensitivity and a durable structure^61^. This means that it is the most advanced conducted material sustainable for solar energy applications.

#### Total density of states (TDOS)

**Fig. 4:**
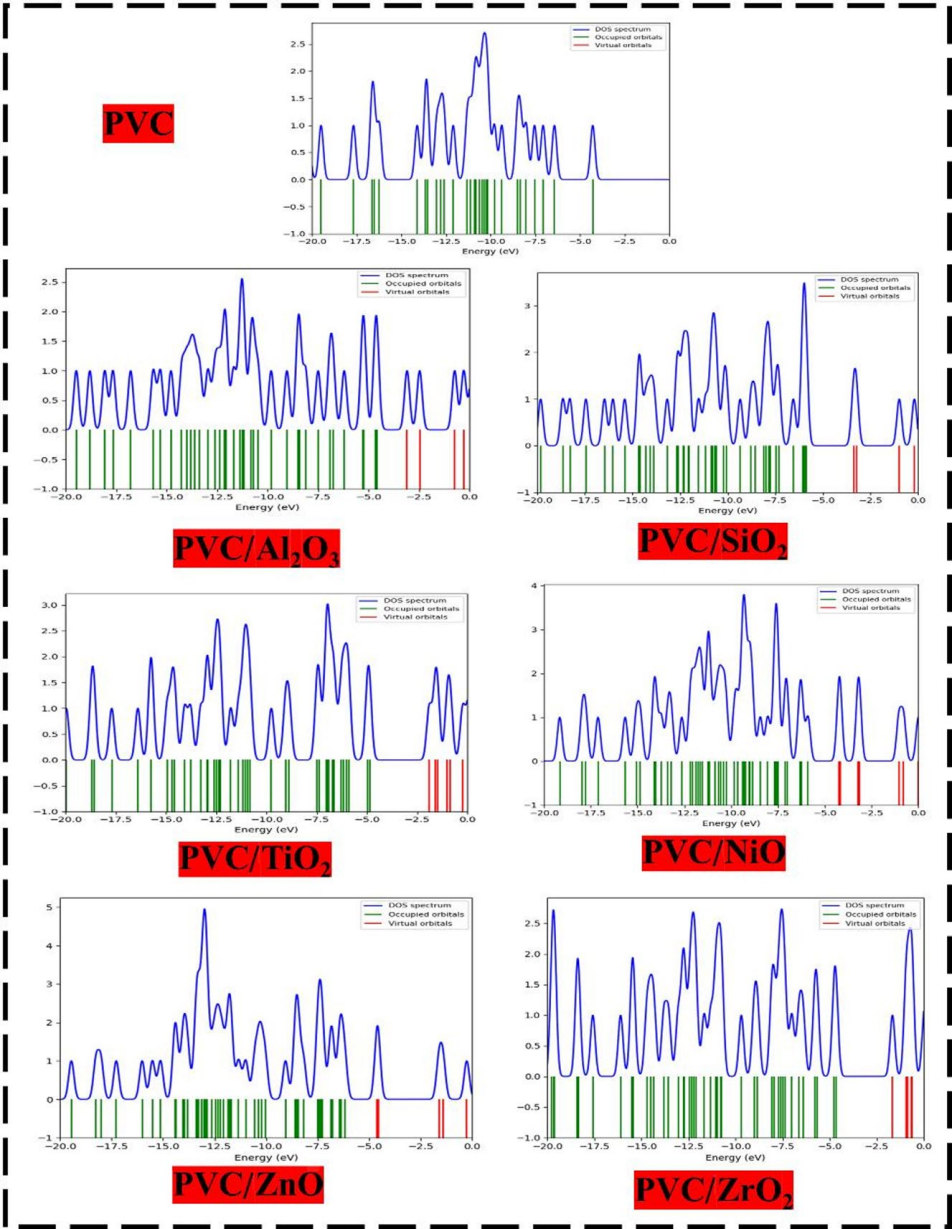
TDOS of PVC and PVC interacted with different metal oxides (Al_2_O_3_, SiO_2_, TiO_2_, NiO, ZnO, and ZrO_2_).

The TDOS serves as a fundamental visualization tool that is crucial for understanding the electrical conductivity of materials and determining their properties. The TDOS provides the quantity of quantum states for each unit energy interval across the entire system. Identifying band gaps, performing a comprehensive analysis of the electrical structure, and examining properties such as conductivity and sensitivity are all critical steps that strengthen the earlier findings related to HOMO-LUMO orbitals and band gap^62^. As shown in Fig. 4, the calculated TDOS for MOs-composites and PVC, which allows for a more in-depth investigation of the changes in electronic properties and intermolecular interactions, is presented. The alteration involving MOs notably lowers the LUMO energy, aligning its levels nearer toward the Fermi level, signifying enhanced interactions among molecules. Due to the extensive electronic transformations caused by molecular orbitals, the PVC matrix exhibited a higher tendency for electron migration, which is reflected in this variation. By incorporating different metal oxides into PVC, such as Al_2_O_3_, SiO_2_, TiO_2_, NiO, ZnO, and ZrO_2_, the LUMO levels are altered, bringing them closer to the Fermi level and enhancing the alignment of the LUMO levels overall. The results concerning the HOMO-LOMO and band gap are supported by this phenomenon, which is particularly pronounced for ZnO and NiO. The ensuing discovery is consistent with our findings, which demonstrated that the changes in electron distribution that occurred as a result of the interaction between PVC and metal oxides led to improvements in charge transfer, electronic characteristics, and conductivity^63^. The aforementioned pattern of changes is crucial for comprehending the responsiveness and stability of the nanocomposite at the molecular level. This further supports previous findings related to HOMO and LUMO orbital distribution and energy, besides the band gap, suggesting that PVC-ZnO may offer enhanced conductivity, reactivity, and structural stability.

#### Partial density of states (PDOS)

**Fig. 5:**
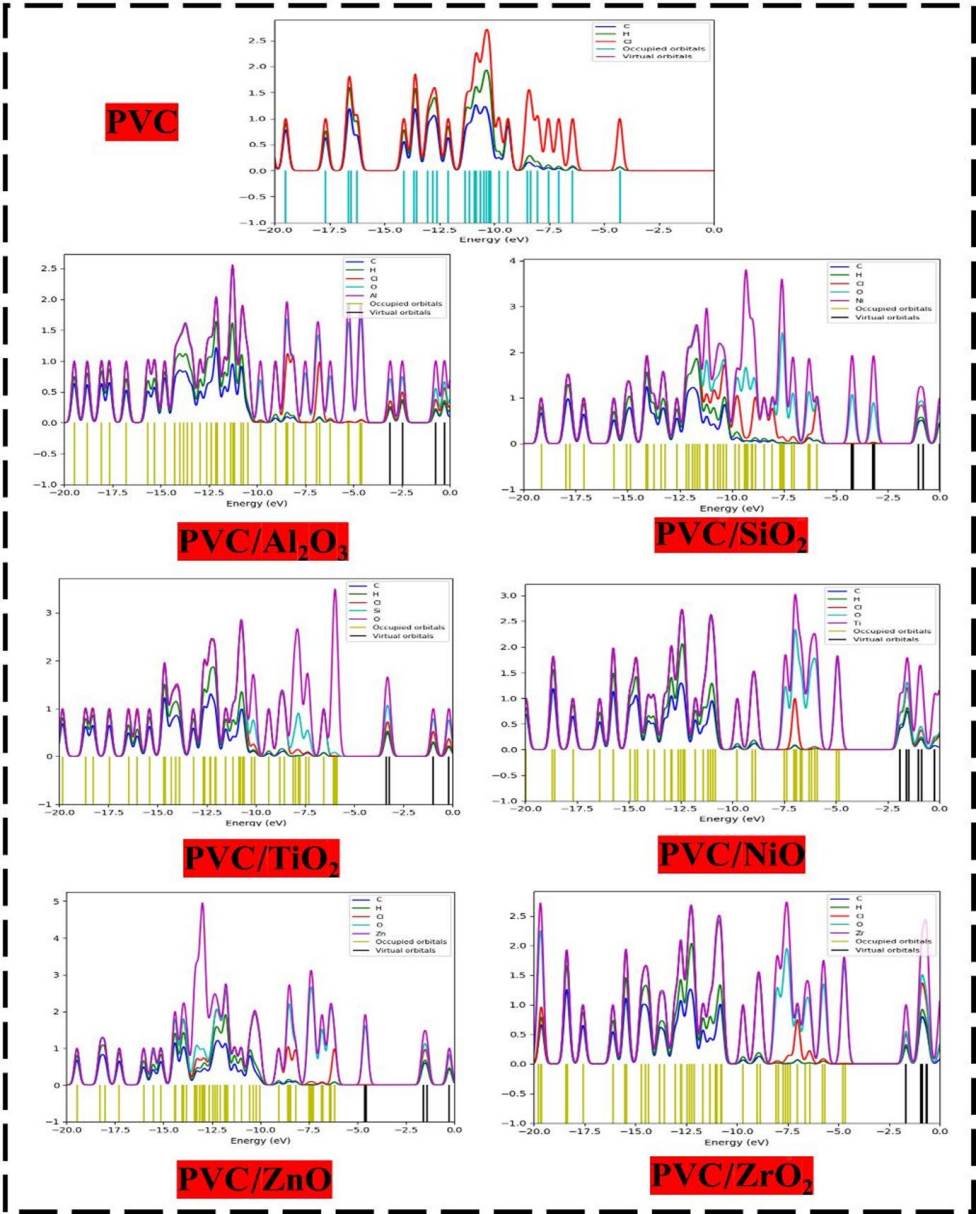
PDOS of PVC and PVC interacted with different metal oxides (Al_2_O_3_, SiO_2_, TiO_2_, NiO, ZnO, and ZrO_2_).

PDOS In order to understand phenomenon like bonding, hybridization, and different electronic interactions, it is helpful to have a holistic picture that shows how individual atomic orbitals or fragments contribute. By showing how different atomic orbitals or fragments (such s, p, and d orbitals) contributed to the TDOS, PDOS gives a holistic view. A better understanding of the atomic orbitals involved in bonding and antibonding interactions can be gained using PDOS. This technique enhances the knowledge of how various components or orbitals impact the electrical structure and features of the substance^53^. The magnitudes of the C-2p, H-1s, O-2p, and Cl-3p orbital patterns show significant changes in the predicted PDOS for PVC new composites, as shown in Fig. 5. The different types of MOs used in the PVC matrix cause the variations in composition. After analyzing the PVC PDOS, it was found that C-2p, H-1s, O-2p, and Cl-3p behave similarly to the HOMO levels in their contributions. Overlapping of the Cl-3p of PVC and the O-2p of molecular orbitals in Al_2_O_3_, SiO_2_, TiO_2,_ NiO, ZnO, and ZrO_2_ indicates a substantial orbital interaction; this leads to an improved configuration of carbon, hydrogen, oxygen, and chlorine atoms across the HOMO and LUMO levels. The combination proves it. This case shows that in order to form physical interactions with molecular orbitals, the PVC molecule gives up a lot of electrons. Because of (Al_2_O_3_, SiO_2_, TiO_2_, NiO, ZnO, and ZrO_2_), an increase in the intensity of PDOS peaks that are dispersed throughout the HOMO and LUMO levels ranging from − 6 to 0 is identified. These peaks are distributed throughout the crystalline structure. This behavior reflects both the coupling that takes place between the Cl-3p atomic orbitals of PVA and the O-2P atomic orbitals of MOs, as well as the energy loss that takes place as a result of MO binding via a Van Dar Waal physical bond. Both phenomena are correlated with one another and clear the interaction and influence of MOs on PVC matrix.

#### Molecular electrostatic potential (MESP)

**Fig. 6:**
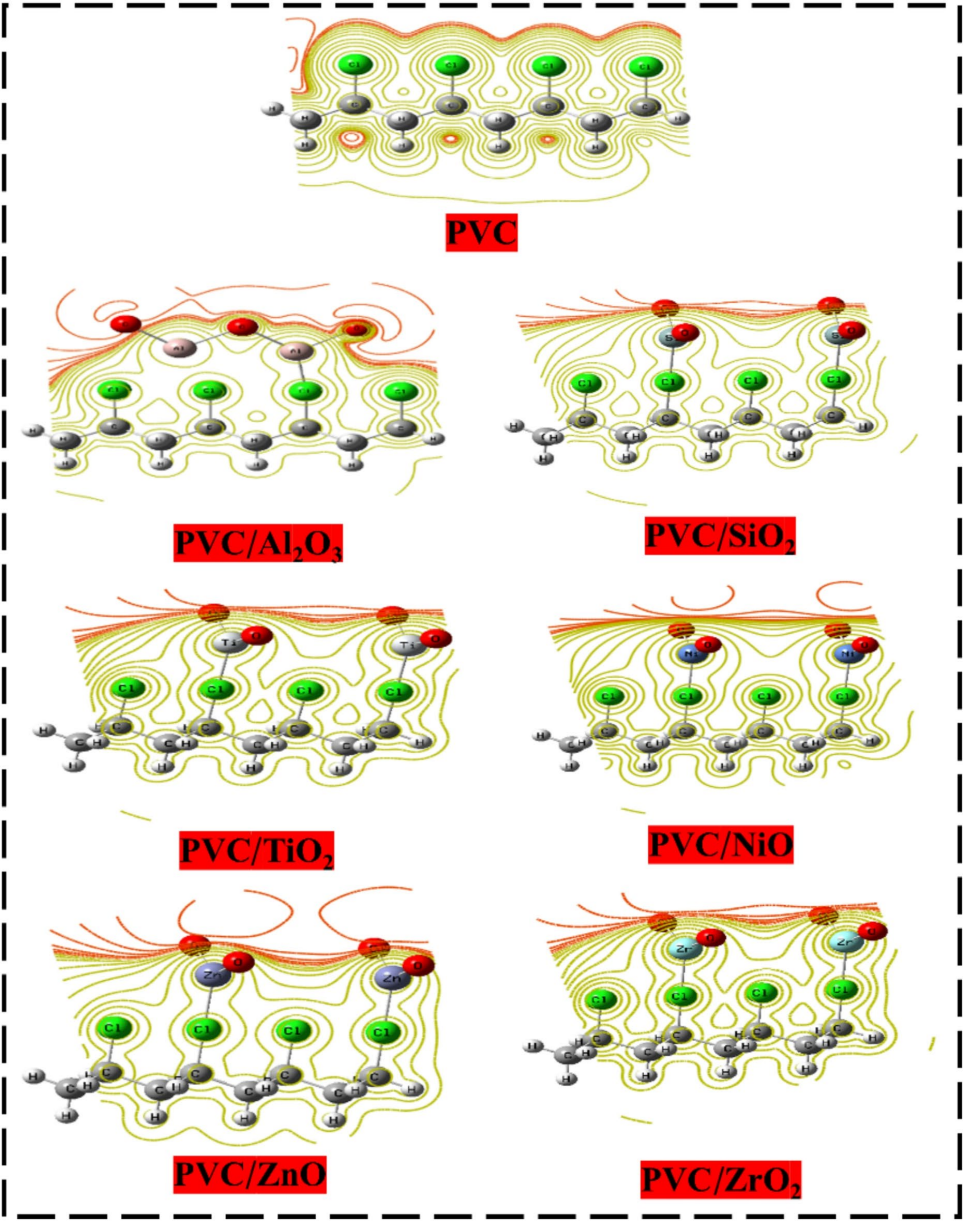
DFT: B3LYP/6–31(d, p) of PVC and PVC interacted with different metal oxides(Al_2_O_3_, SiO_2_, TiO_2_, NiO, ZnO, and ZrO_2_) contour MESP map.

Besides, the MEP contour map as a graphic representation is an essential analysis which demonstrates the electrostatic potential display around a molecule. The ability to see regions of both negative and positive charge is vital for understanding the connections and sensitivity of molecules, and they provide assistance in both of these regions^64^. The potential energy correlated to a particular charge, as measured at various points around a molecule, is shown by the MEP. This property is determined by the configuration of the electrons as well as nuclei within the molecule. The MEP contours appear via surfaces or as lines that connect locations with the same electrostatic potential^65^. In doing so, they pinpoint potential targets of electrophilic or nucleophilic sides. Red and orange appearing on the MEP map indicate locations with a very negative electrostatic potential (plenty in electrons), while yellow indicates areas with a very positive electrostatic potential (deficient in electrons)^66^. The significance of employing MESP primarily lies in its application for examining structure sensitivity, reactivity, and stability. The MESP of PVC, along with its reactions with various metal oxides such as Al_2_O_3_, SiO_2_, TiO_2_, NiO, ZnO, and ZrO_2_, has been analyzed, as illustrated in Fig. 6. The red areas typically have the most potential, while the yellow lines frequently possess the lowest potential. The red regions represent the highest potential. After further investigation, it was shown that the Cl atom is the core of the active PVC reactivity^58^. The red color redistributed and concentrated around the MOs –O– atoms when PVC interacted with various MOs. This demonstrated that PVC’s reactivity increased, and that MOs strengthened the active sides of PVC. On account of this, the electrical characteristics of PVC have improved, and it is now capable of being utilized in a wide variety of electrical and optical applications.

#### Physical, reactivity and stability parameters

Intended for additional investigation and analysis of the reactive characteristics and durability of composite substances, particularly important chemical reactivity variables were identified for all composites. These parameters are fundamental for explaining the durability of structures, sensitivity developments, molecular manners, physical and chemical changes, and electrical characteristics due to their informative significance^54^. Table 2 displays the results of the analyses of the descriptors for PVC-MOs composites, which include ionization potential (IP), electronegativity (χ), electron affinity (EA), softness (σ), hardness (η), and electrophilic (ω).

**Table 2:** Calculated physical, stsability and reactivity characteristics of PVC and PVC interacted with different metal oxides using DFT: B3LYP/6–31(d, p).

Parameters	PVC	PVC–Al_2_O_3_	PVC-SiO_2_	PVC-TiO_2_	PVC-NiO	PVC-ZnO	PVC-ZrO_2_
*E*_*HOMO*_	–4.3090	–4.8741	–5.9310	–4.9781	–5.7316	–6.0516	–4.8086
*E*_*LUMO*_	1.1701	–3.4551	–3.7027	–2.1024	–4.4507	–4.7487	–1.8060
IP	4.3090	4.8741	5.9310	4.9781	5.7316	6.0516	4.8086
EA	–1.1701	3.4551	3.7027	2.1024	4.4507	4.7487	1.8060
χ	1.5695	4.1646	4.8169	3.5403	5.0912	5.4002	3.3073
η	3.0050	0.7095	1.1142	1.1742	0.6405	0.6515	1.5013
σ	0.3328	1.4094	0.8975	0.8516	1.5613	1.5349	0.6661
ω	0.4099	12.2226	10.4122	5.3371	20.2344	22.3808	3.6429
*ε*	2.4396	0.0818	0.0960	0.1874	0.0494	0.0447	0.275

Equation 3 and Eq. 4 were used to compute the ionization potential (IP) and electron affinity (EA), two crucial quantities, with the help of the E_HOMO_ and E_LUMO_, which are highly dependent on these factors. An alternative view holds that the IP is the possible energy needed to eliminate electrons from a molecule, resulting in the generation of free radicals. Conversely, EA is defined as the energy necessary to attract electrons to the molecule, leading to the formation of negative ions that enhance the organism’s nucleophilicity^40^. In addition, electronegativity (χ) is indicative of the dispersion of electrons inside substances. Readings that suggest an increased electronegativity imply a higher electronegativity, which in turn increases the substance’s reactivity and its sensitivity. When compared to the other composites that were investigated, PVC-ZnO exhibited the greatest electronegativity, with a value of 5.4002 eV overall. This demonstrates that they have an exceptional responsiveness, as demonstrated by the band gap data as well as the MESP result. The chemical hardness (η) of a substance is a mathematical measure that measures its resistance to structural distortion or charge splitting^67^. On top of that, softness (σ) is generally thought to be the opposite of hardness (η), which is a crucial feature for the sensitive properties of materials^68^. Having the greatest softness rating merged with the smallest hardness rating suggests that the composite is more durable and stable. As a result, the material is perfectly suited for use in electrical and optical devices^69^. The most remarkable softness ratings were achieved by this very reactive chemical, which was PVC-ZnO because of its enhanced sensitivity and durability features. Ultimately, a substance’s electrophilicity (ω) and nucleophilicity (ε) are the defining characteristics of its molecular reactivity. These two attributes dictate the likelihood of a substance accepting or donating electrons from its environment^43^. Quantifying the capacity of a substance to gather up electrons provides the electrophilicity (ω) number. On the other hand, the nucleophilicity (ε) importance, regarded as the inverse of electrophilicity (ω), reflects the capacity of a substance to give away or transport electrons in association with a certain chemical composition^70^. Substances are classified into three groups based on their electrophilicity: strong electrophiles, which have an ω value of (1.5 eV) or higher, medium electrophiles, which have an ω value of (0.8 to 1.5 eV), and low electrophiles lower than 0.8 eV^71^. PVC is classified as a low electrophile. Through the incorporation of molecular orbitals, the electrophilicity of the composites is enhanced, resulting in the formation of highly resistant electrophiles, particularly between PVC and ZnO. This provides evidence that the surface area responsiveness and absorption capacities of the PVC matrix were improved by the addition of ZnO NPs by the PVC matrix^52^. The results referring to MESP and band gap demonstrated that PVC-ZnO possessed higher sensitivity and reactivity. This highlights the fact that PVC-ZnO is an extremely useful compound for electrical and optical applications, in addition to proving the effects of composition.

#### DFT study of PVC-ZnO hybridization with G

**Fig. 7:**
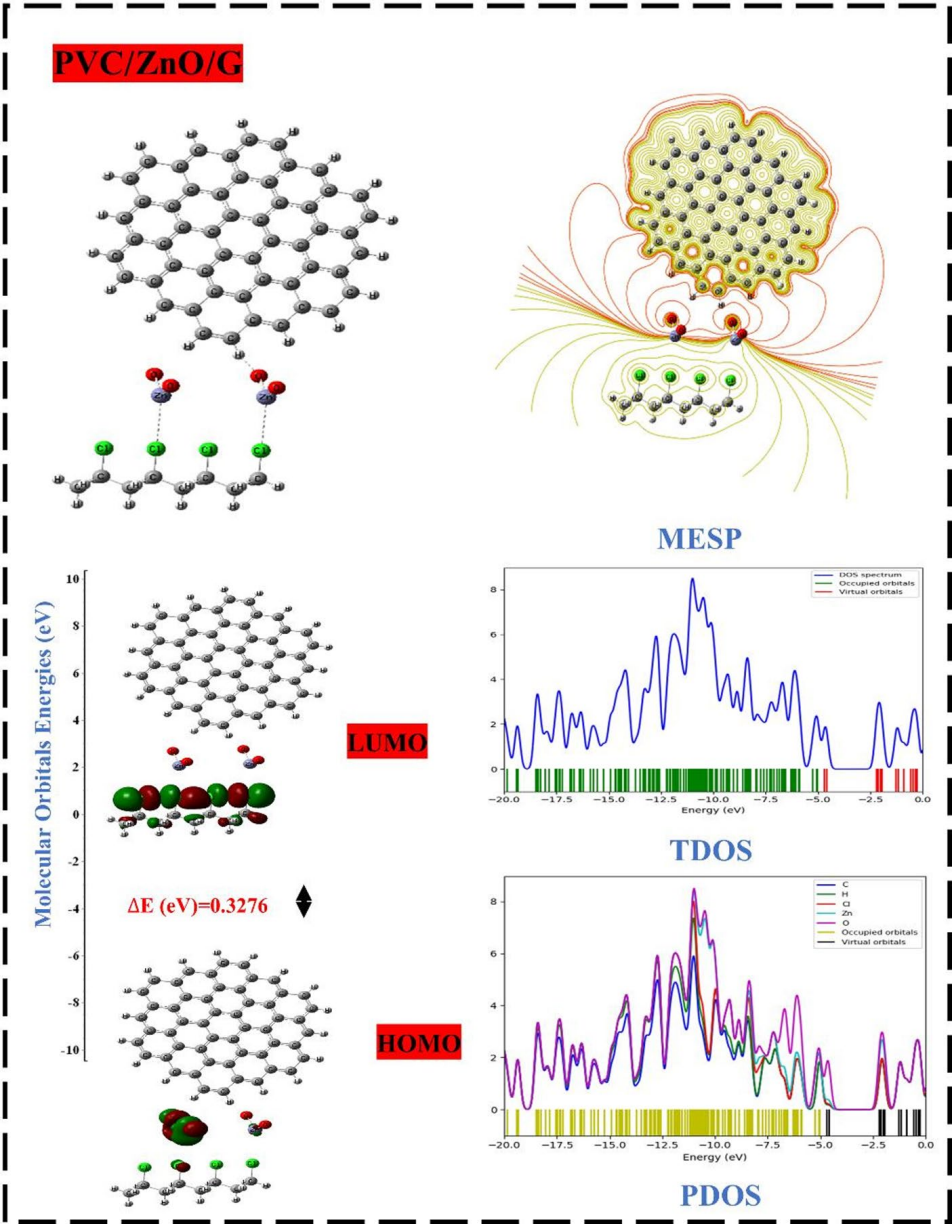
DFT: B3LYP/6–31(d, p) calculations for optemized structure and HOMO/LUMO orbital distrbution and MESP as contour TDOS and PDOS of the PVC/ZnO interactions with G.

Graphene, a commonly prevalent 2D layer, provides outstanding distinctive properties that contribute to improved efficiency due to its physicochemical surface area, specific capacitance, thermal stability, mechanical, and electrical features^32^. Having a pore size inside the G sheet substantially influences its characteristics and applications in various industries^72^. These nanomaterials demonstrate significant potential for various applications such as electronic devices, energy storage, as well as in optoelectronic devices including light-emitting diodes (LEDs), catalysis, photosensors (PS), thin-film transistors (FET), and solar cells (SC)^12^. Consequently, the hybridization with G nanosheets serves as a robust reinforcement strategy that increases the porosity, surface area, conductivity, beside thermal and physicochemical stability of the material, thereby improving electron transfer through the defects, which reflects its affinity for charge transfers and/or conductivity characteristics^73^. Consequently, PVC/MO/G was developed to integrate the superior electron transport ability and/or electrical and optical properties of PVC/MO boosted structure by adapting the bandgap influenced by the G ingredient. The proposed structures were also analyzed to provide HOMO/LUMO orbital distributions and MESP mapping. The previous investigation demonstrated that PVC/ZnO exhibited both chemical stability and activity as a structure^51^. So, the proposed interaction of PVC/ZnO with G sheet is recommended for further study, as illustrated in Fig. 7. The interaction of G was evaluated with the most electrically enhanced structure, PVC/ZnO. The localization of the HOMO orbitals on ZnO is the result of the interaction between G and PVC/ZnO with ZnO through the –O– atom. On the other hand, the LUMO orbitals are dispersed along the PVC chain^74^. Consequently, this results in a large decrease in the gap that separates them (0.3276 eV), which is indicative of a major improvement in conductivity and reactivity. This conclusion is strengthened by the result of TDOS, which revealed a considerable shift in HOMO levels close to the Fermi level, ranging from − 6.0516 to -5.0380 eV, in addition to a redistribution to the levels that were intense. This fluctuation was accompanied by a major change in the difference between HOMO and LUMO levels^52^. As a result of the massive molecular interactions that occurred between PVC, ZnO, and G, LUMO levels emerged, and the band gap sharply decreased. Additionally, the contour MESP map showed that the strong intense red line areas originated from the –O– atom of ZnO and extended around the edge of the G sheet^75^. This enhanced the edge network of the G sheet, which contributed to the increased reactivity and conductivity of the PVC matrix. In the meantime, the calculated PDOS for the hybrid composite PVC/ZnO/G revealed that the integration of ZnO and G influence into the PVC produced a variation in the configuration of C-2p, H-1s, O-2p, and Cl-3p orbitals. This variation resulted in an elevation of the HOMO and LUMO levels, causing an overlap of O, Zn, and Cl across these levels, along with an increase in the O-2p magnitude. The interaction between ZnO and G with PVC leads to a notable reduction in charges, attributed to the interactions with PVC (Cl-3p orbitals), indicating weak interactions. The establishment of this arrangement is crucial for comprehending the transformation of PVC/ZnO/G in terms of reactivity, stability, and long-term viability. All results indicate an increased number of electrons due to the modifications induced by the interactions among PVC, ZnO, and G. This conclusion aligns with prior research findings regarding HOMO/LUMO sistrbution, band gap energy, TDOS and contour MESP map, indicating improvements in electrical characteristics, durability, and stability.

For further comparison and modification tracing the hybridization with ZnO and G effect on the PVC matrix, the reactivity and stability properties such as TDM, band gap energy, electrophilicity, electronegativity, hardness, and softness were calculated and compared with previously calculated PVC/ZnO parameters, as presented in Table 3. The outcome of PVC/ZnO/G demonstrated a notable reduction in the band gap, reaching 0.3276 eV, accompanied by a minor alteration in TDM, indicating a substantial improvement in conductivity. The results demonstrate the significant impact of G on the PVC/ZnO composite, resulting in a reduction of the band gap, which subsequently enhances transport of electrons, responsiveness, and electrical conductivity^76^. Additionally, the hybridization enhanced significantly the electrophilicity of PVC/ZnO, in addition to improving and changing the composite into materials that are highly conductive. Additionally, there was an improvement in softness, which implies that there was an increase in stability. According to the findings, the addition of G sheets results in an increase in surface area that is in accordance with the porous structure^77^. This, in turn, leads to an improvement in the responsiveness and electrical conductivity of PVC, as well as its stability. The findings that were connected to the band gap and the contour MESP map were validated by the overall reactivity values^78^. These values indicated that PVC/ZnO/G is a highly reactive, conductive, and stable composite due to its specific electronic properties. The functionality and dependability of electronics composed of polymer nanocomposites are greatly affected by the manufacturing faults, including induced porosity^79^. So, this work has investigated a new hybrid composite PVC-ZnO-G in great detail. The combination of surface area and porosity provides the composite excellent electrical and optical characteristics, making it ideal for use in solar active cells.

**Table 3:** Calculated reactivity and stability characreristics using B3LYP/LANL2DZ of the PVC/ZnO interactions with G.

Parameters	PVC/ZnO/G
TDM	12.3859
∆E_g_	0.3276
*E*_*HOMO*_	–5.0380
*E*_*LUMO*_	–4.7103
IP	5.0380
EA	4.7103
χ	4.8742
η	0.1639
σ	6.1013
ω	72.4766
*ε*	0.01380

### Experimental investigation of PVC/ZnO/G nanocomposites

#### XRD & FTIR analysis

**Fig. 8:**
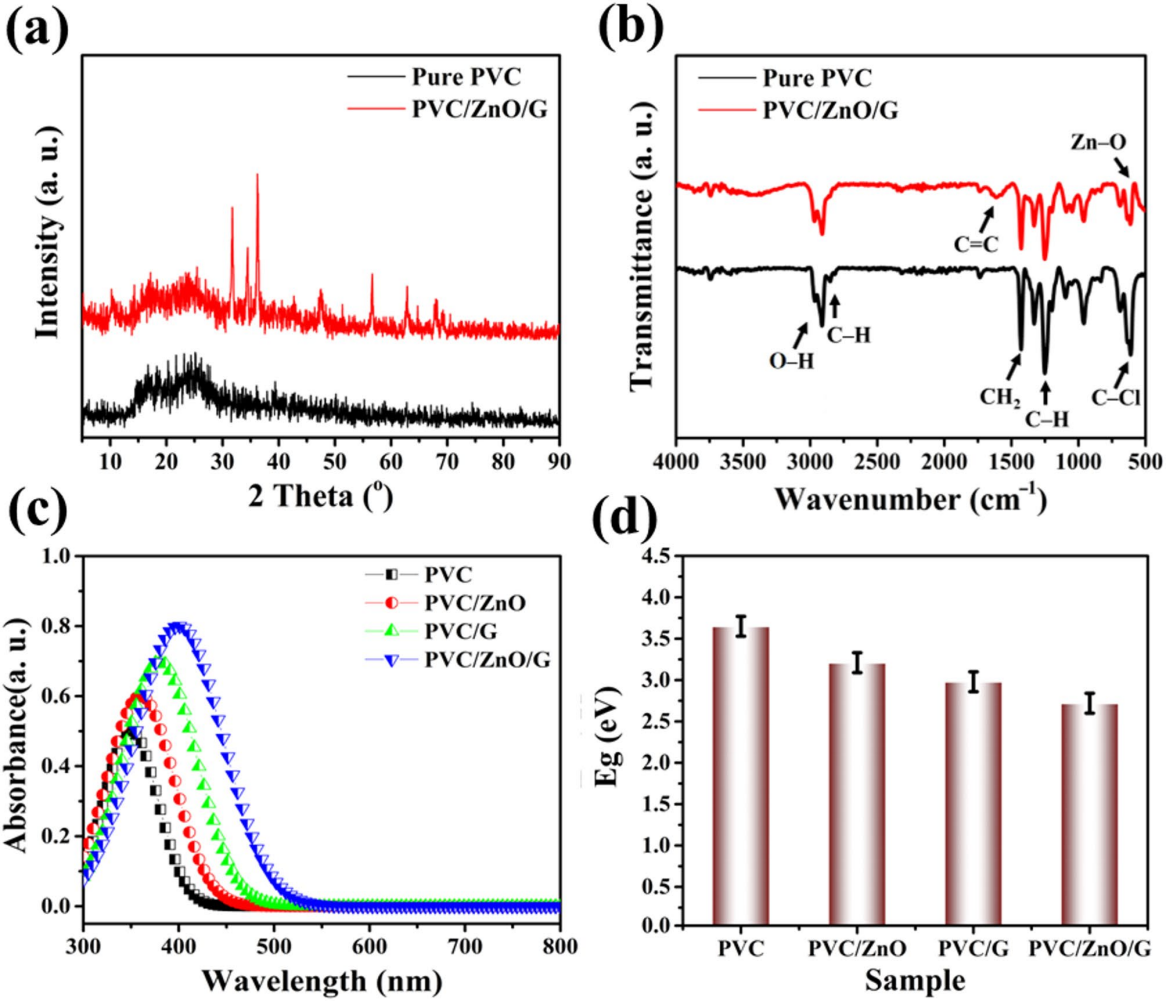
(**a**) XRD diffraction patterns, (**b**) FTIR spectra, (**c**) UV–Vis absorbance spectra, and (**d**) Tauc plots for the energy band gap determination of pure PVC, PVC/ZnO, PVC/G, and PVC/ZnO/G composites.

The XRD patterns of pristine PVC and PVC/ZnO/G composites reveal distinct structural differences that confirm the successful integration of ZnO NPs and graphene into the polymer matrix, as seen in Fig. 8a. Pristine PVC displayed a broad, low-intensity amorphous halo centered at approximately 2θ ≈ 20^°^, characteristic of its predominantly amorphous structure with only minor short-range ordering of the vinyl chloride chains. This absence of sharp crystalline reflections indicates a lack of long-range periodicity in the neat polymer, consistent with earlier reports on halogenated polymer matrices. By contrast, the PVC/ZnO/G composite exhibited sharp and well-defined diffraction peaks superimposed on the amorphous halo, confirming the crystalline nature of the embedded ZnO NPs. The prominent reflections located at 2θ ≈ 31.7^°^, 34.4^°^, 36.2^°^, 47.5^°^, 56.6^°^, 62.8^°^, and 67.9^°^ were indexed to the (100), (002), (101), (102), (110), (103), and (112) planes of the hexagonal wurtzite phase of ZnO, respectively, in excellent agreement with JCPDS card No. 36-1451. These assignments align with classical structural data on ZnO, as summarized by Z. L. Wang (2004)^80^, which reviews the characteristic wurtzite peaks and properties of ZnO nanostructures. The absence of any secondary peaks corresponding to Zn(OH)_2_, ZnCl_2_, or other impurity phases confirms the high phase purity of the synthesized composite^81^. In addition to ZnO reflections, the baseline of the PVC/ZnO/G diffractogram showed a slight elevation at low angles, which can be attributed to graphene incorporation. Although graphene typically exhibits a broad (002) reflection at 2θ ≈ 25–26^°^, its weak and diffuse nature in polymeric nanocomposites often merges with the polymer halo, suggesting homogeneous dispersion of graphene sheets within the PVC matrix^82^. The crystallite size of ZnO in the composite was estimated using the Scherrer equation applied to the most intense (101) reflection^83^. The calculated size fell within the nanometer range (≈ 22.7 nm).

Notably, slight peak shifts of ZnO reflections toward lower 2θ angles were observed compared to the standard JCPDS values, signifying lattice expansion. This behavior is commonly attributed to polymer-induced tensile stress and the presence of oxygen vacancies at the ZnO surface, both of which are known to enhance catalytic activity^84^. The broadening and intensity modulation of the diffraction peaks also suggest excellent dispersion of ZnO nanocrystals and graphene within the PVC matrix, preventing aggregation and facilitating large interfacial contact areas^85^. Collectively, the XRD analysis confirms the successful formation of a hybrid architecture in which crystalline ZnO NPs are uniformly dispersed in the amorphous PVC matrix and electronically coupled with graphene sheets^47^. This structural configuration provides abundant catalytic sites, promotes defect-mediated charge transfer, and establishes conductive pathways—structural features that directly correlate with the enhanced optical absorption, reduced band gap, and superior electrochemical performance of the PVC/ZnO/G CEs in DSSCs.

Figure 8b compares the FTIR spectra of pristine PVC and the PVC/ZnO/G composite, revealing key structural modifications induced by the incorporation of ZnO NPs and graphene nanosheets. In pure PVC, characteristic vibrational signatures were clearly observed: the stretching vibrations of C–H bonds appeared at 2914 and 2970 cm^− 1^, while bending vibrations of CH_2_ and C–H in CHCl were detected at 1426 and 1264 cm^− 1^, respectively. Additionally, the stretching mode of the C–Cl bond was evident at 608 cm^− 1^, which is a well-established fingerprint of PVC’s halogenated backbone^31^. Upon hybridization with ZnO and graphene, noticeable shifts and the emergence of new absorption features were recorded, confirming strong interfacial interactions between the fillers and the polymer chains. The intensity reduction and slight displacement of the C–Cl band suggest perturbation of the PVC matrix due to ZnO surface interactions, consistent with the formation of coordination bonds and partial dehydrochlorination processes^86^. Simultaneously, graphene incorporation introduced a distinct absorption band near 1596 cm^− 1^, which corresponds to skeletal C = C stretching, validating the integration of sp^2^-hybridized carbon networks within the composite.

Most notably, the PVC/ZnO/G spectrum displayed a new vibrational feature at 515 cm^− 1^, attributable to the stretching vibration of Zn–O bonds, which is a strong indicator of the presence of ZnO NPs^71^. The coexistence of Zn–O, C–Cl, and C = C vibrations within a single spectrum highlights the formation of a multifunctional hybrid system in which ZnO contributes catalytic activity and oxygen-containing surface chemistry^47^, while graphene provides conductive networks and structural reinforcement. These spectral modifications not only confirm the successful fabrication of the PVC/ZnO/G nanocomposite but also elucidate the molecular-level interactions that govern its enhanced optoelectronic and electrochemical properties. The suppression of PVC’s halogen vibrational intensity, coupled with the emergence of Zn–O and C = C signatures, provides compelling evidence of synergistic interfacial coupling between the polymer host and nanofillers^85^. Such interactions are directly responsible for the improved charge transport, catalytic behavior, and stability observed in the photovoltaic performance of the PVC/ZnO/G CEs.

#### Optical properties: absorbance and band gap analysis

The absorbance spectra of the investigated CEs provide critical insights into the light-harvesting ability and optical response of pristine PVC and its composites with ZnO and graphene, as seen in Fig. 8c. Pure PVC exhibits a relatively narrow absorption profile centered in the UV region (around 330–370 nm), consistent with its wide-bandgap insulating nature. The limited spectral coverage restricts its capacity to efficiently utilize the visible region of the solar spectrum, thereby diminishing its suitability for optoelectronic applications. Upon incorporation of ZnO NPs, a noticeable red-shift in the absorption edge is observed, extending the absorption into the visible range (~ 420 nm). This enhancement is attributed to the semiconducting nature of ZnO, whose intrinsic band structure introduces defect levels and oxygen vacancies that improve visible light absorption while simultaneously acting as active sites for interfacial interactions with PVC^87,88^. Further incorporation of graphene markedly transforms the absorbance profile, producing a broad and continuous absorption spectrum across the 300–800 nm region. Graphene’s zero-bandgap and π–π* transitions introduce delocalized electronic states, effectively enhancing light–matter interactions and suppressing exciton recombination^89^. The synergistic effect becomes most evident in the ternary PVC/ZnO/G composite, which demonstrates the highest and broadest absorbance intensity, spanning from the UV region into the visible domain. This broadening directly corresponds to a greater ability to harvest incident photons and generate excitons, which is a crucial prerequisite for achieving higher photocurrent densities in DSSCs. The collective role of ZnO as a visible-light sensitizer and graphene as a charge delocalization medium provides a dual mechanism: enhanced photon absorption and improved charge extraction^90^. This trend is in excellent agreement with the photovoltaic data, where higher J_sc_ values are recorded for ZnO- and graphene-containing composites, particularly PVC/ZnO/G.

The optical band gap values derived from Tauc plots further validate the structural and electronic modifications induced by ZnO and graphene doping, as displayed in Fig. 8d. Pure PVC exhibits the largest band gap of ~ 3.65 eV, typical of wide-bandgap insulating polymers with limited electron mobility. The high band gap hinders visible light absorption and electron excitation, which explains the weak absorbance and poor photovoltaic performance of pristine PVC. With ZnO incorporation, the band gap narrows to ~ 3.21 eV, attributed to the introduction of localized defect states and enhanced polymer–semiconductor coupling, which facilitates electronic transitions at lower photon energies^91^. This bandgap reduction supports the modest improvement in both absorbance intensity and device efficiency observed in PVC/ZnO composites^33^. The inclusion of graphene further reduces the band gap to ~ 2.98 eV, owing to the strong electronic coupling between PVC chains and graphene’s delocalized π-electrons^88^. Graphene not only contributes additional electronic states within the forbidden gap but also enables effective charge delocalization, thus lowering the excitation threshold. The most significant band gap narrowing occurs in the ternary PVC/ZnO/G system (~ 2.72 eV), underscoring the synergistic role of ZnO defect states and graphene’s extended π-conjugation in reshaping the electronic structure of the host polymer^92^. This narrowed band gap enables absorption of lower-energy photons within the visible region, translating directly into higher photocurrent generation, enhanced IPCE values, and improved overall DSSC efficiency. Therefore, the optical results demonstrate a consistent and complementary trend: progressive broadening of the absorbance spectrum and systematic narrowing of the band gap with ZnO and graphene incorporation^93^. These modifications are central to optimizing DSSC performance, as they simultaneously enhance light harvesting and facilitate charge carrier excitation and transport^94^. More importantly, the findings confirm the predictive outcomes of DFT modeling, which identified ZnO and graphene as optimal dopants for synergistically tailoring PVC’s electronic and optical characteristics. Thus, the PVC/ZnO/G composite emerges as a multifunctional CE material, bridging the gap between cost-effectiveness and high performance, while offering a blueprint for the rational design of polymer–metal oxide–graphene nanostructures for next-generation solar energy conversion technologies.

#### Surface morphology and pore structure analysis

**Fig. 9:**
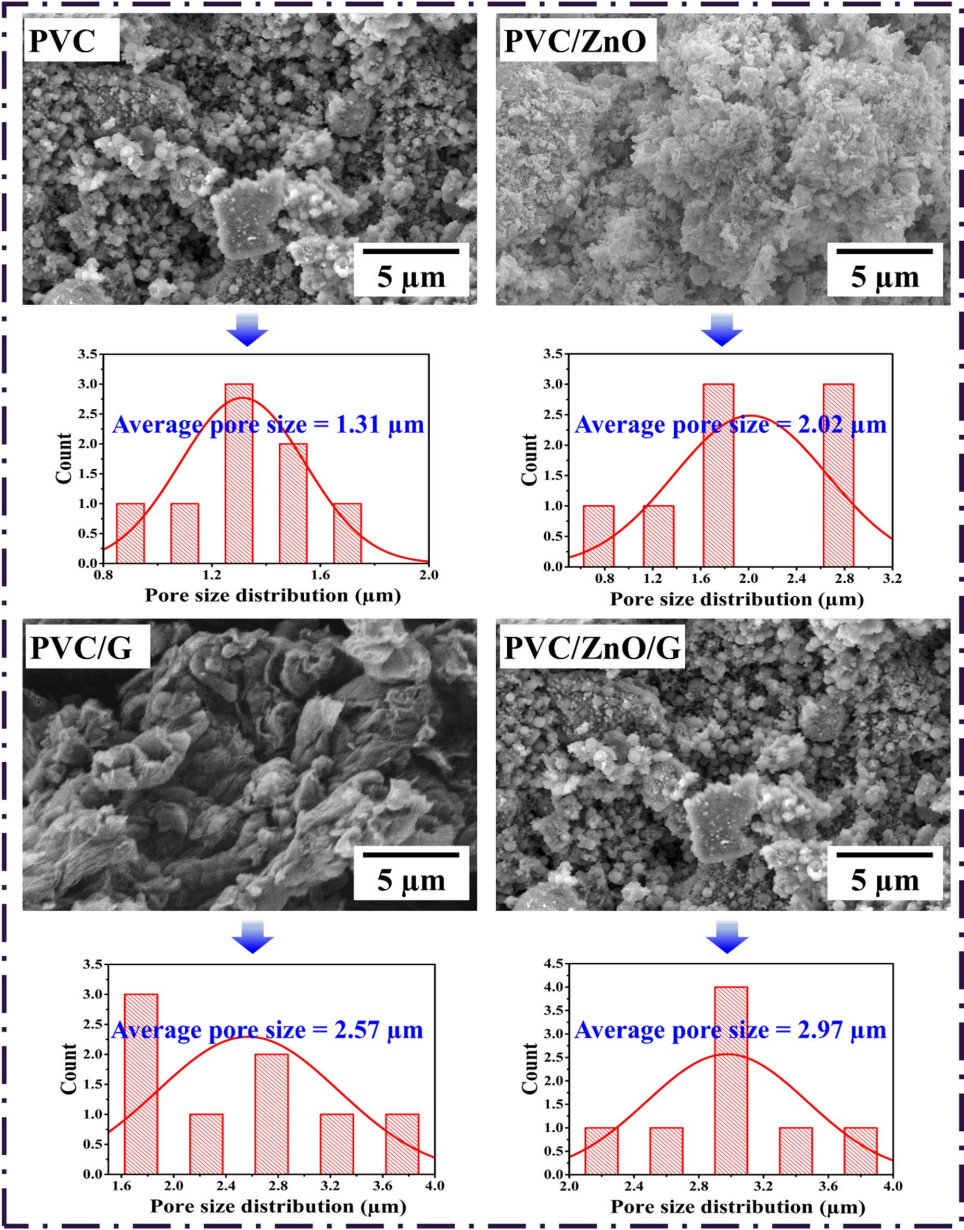
SEM micrographs of PVC-based CEs modified with different metal oxides, highlighting the progressive structural evolution, pore formation, and surface modifications induced by MO incorporation. The corresponding pore size distribution histograms, obtained from ImageJ analysis, further illustrate the influence of MO type on the microstructural characteristics of the composites.

The surface morphology and pore characteristics of CEs play a critical role in determining their electrochemical activity and photovoltaic efficiency, as they directly influence electrolyte penetration, ion diffusion, and the accessibility of catalytic sites. Statistical pore size analysis, carried out using ImageJ software, revealed systematic variations in pore dimensions across the investigated samples. The average pore sizes were determined to be 1.31 μm for pristine PVC, 2.02 μm for PVC/ZnO, 2.57 μm for PVC/G, and 2.97 μm for the ternary PVC/ZnO/G composite, as seen in Fig. 9. The relatively small pore size in pristine PVC indicates a compact and dense morphology, consistent with its insulating character and poor ionic permeability^95^. Such a structure limits electrolyte infiltration and restricts the number of accessible electroactive sites, thereby contributing to the high R_ct_ and the modest photovoltaic performance (η = 4.70%). Upon incorporation of ZnO NPs, a significant increase in pore size (2.02 μm) was observed. This enlargement is attributed to the heterogeneous dispersion of ZnO within the PVC matrix, which disrupts polymer chain packing and induces microstructural voids^9^. The resulting porous architecture facilitates better penetration of the electrolyte and enhances the effective catalytic interface, as reflected in the improved J_sc_ and efficiency. Graphene incorporation yielded an even larger pore size of 2.57 μm, which can be ascribed to the two-dimensional lamellar morphology of graphene nanosheets^7^. These sheets act as spacers within the polymer matrix, preventing excessive compaction of PVC chains and establishing interconnected pore networks. Such a morphology promotes efficient diffusion of ${\mathrm{I}}_{3}^{-}/{\mathrm{I}}^{-}$ species, reduces mass transport limitations, and enhances charge transport across the CE. The consequence is evident in the increased J_sc_ and efficiency observed for PVC/G electrodes.

The most pronounced improvement was achieved in the ternary PVC/ZnO/G composite, where the average pore size reached 2.97 μm. The synergistic interaction between ZnO and graphene disrupts the polymer matrix more effectively than single-component fillers, yielding a highly porous structure with well-connected pathways for electrolyte infiltration^96^. Larger and interconnected pores not only enhance the diffusion kinetics of redox species but also provide an expanded electrochemically active surface area for catalytic reactions. This microstructural advantage correlates strongly with the lowest R_ct_ among the polymer-based electrodes and the highest efficiency, approaching that of the Pt reference electrode. These findings underscore the critical role of morphology–property relationships in dictating CE performance. While PVC alone provides a dense, resistive surface, the progressive incorporation of ZnO and graphene induces hierarchical porosity that simultaneously supports catalytic activity and charge transport^97^. The ternary hybrid thus embodies an optimized structural framework that balances conductivity, catalytic site availability, and electrolyte accessibility^98^. This demonstrates that tailoring the pore structure through rational nanofiller integration is a viable strategy for designing multifunctional, cost-effective CEs that rival noble metals in DSSCs.

#### Surface roughness, porosity and electrical conductivity

The surface roughness, porosity, and electrical conductivity of the PVC-based composite CEs are key parameters governing interfacial charge transfer and redox kinetics in DSSCs. The consolidated results are summarized in Table 4 and reveal a clear structure–property–performance correlation induced by ZnO and graphene incorporation. Pristine PVC exhibited the lowest average surface roughness (Ra = 5.8 μm), apparent porosity (18.7%), and electrical conductivity (18 S/m), indicating a relatively dense and smooth morphology. Such characteristics limit the effective electrode–electrolyte contact area, restrict electrolyte penetration, and reduce the density of accessible catalytic sites for the ${\mathrm{I}}^{-}/{\mathrm{I}}_{3}^{-}$ redox reaction, thereby constraining charge-transfer efficiency.

Upon incorporation of ZnO nanoparticles, noticeable improvements were observed. The Ra increased to 6.4 μm, accompanied by an increase in apparent porosity to 20.2% and an enlarged average pore size of 2.02 μm. These changes suggest that ZnO acts as a microstructural modifier by disrupting polymer chain packing and generating additional microvoids within the PVC matrix^23^. Consequently, electrolyte infiltration is facilitated and the number of active catalytic sites is increased. In parallel, electrical conductivity improved to 31 S/m, reflecting the semiconducting nature of ZnO and its contribution to enhanced electron percolation pathways^99^. However, the conductivity enhancement remained moderate, indicating that ZnO alone cannot fully overcome the intrinsic insulating nature of PVC. A more pronounced effect was achieved with graphene incorporation^100^. The PVC/G composite exhibited a higher surface roughness (Ra = 7.1 μm), increased apparent porosity (21.8%), and a larger average pore size of 2.57 μm. The two-dimensional morphology of graphene sheets promotes the formation of interconnected pore networks and microchannels, which enhance electrolyte diffusion and reduce mass-transport limitations at the CE/electrolyte interface^101^. Moreover, graphene dramatically boosted electrical conductivity to 57 S/m due to its high intrinsic carrier mobility and extended π-conjugated network, confirming its dominant role in facilitating efficient charge transport while simultaneously increasing the density of catalytically active surface features^102^.

The most significant enhancement was observed for the PVC/ZnO/G hybrid composite, which exhibited the highest surface roughness (Ra = 8.5 μm), apparent porosity (23.1%), average pore size (2.97 μm), and electrical conductivity (66 S/m). The synergistic coexistence of ZnO nanoparticles and graphene sheets results in a hierarchical porous architecture, where ZnO acts as a spacer and anchoring agent that prevents graphene restacking, while graphene establishes continuous and highly conductive networks throughout the composite^31^. This cooperative interaction maximizes electrolyte accessibility, accelerates ion diffusion, and ensures efficient charge collection and transport^103^. Therefore, the progressive enhancement following the sequence PVC < PVC/ZnO < PVC/G < PVC/ZnO/G clearly demonstrates that simultaneous engineering of surface roughness, porosity, and electrical conductivity is essential for optimizing CE performance^8^. Increased surface roughness and porosity promote electrolyte penetration and catalytic reaction kinetics, while improved conductivity ensures rapid electron transport^82^. Together, these structural and electrical modifications underpin the superior electrochemical activity and photovoltaic performance achieved by the PVC/ZnO/G composite CEs.

**Table 4:** Consolidated values of average pore size, surface roughness, apparent porosity, and electrical conductivity for pristine and MO-enriched PVC-based CEs employed in DSSCs, highlighting the structural and electrical modifications induced by ZnO and graphene incorporation.

Sample	Average pore size (µm)	Average surface roughness, Ra (µm)	Apparent porosity (%)	Electrical conductivity (S/m)
PVC	1.31	5.8	18.7	18
PVC/ZnO	2.02	6.4	20.2	31
PVC/G	2.57	7.1	21.8	57
PVC/ZnO/G	2.97	8.5	23.1	66

#### EIS observations

**Fig. 10:**
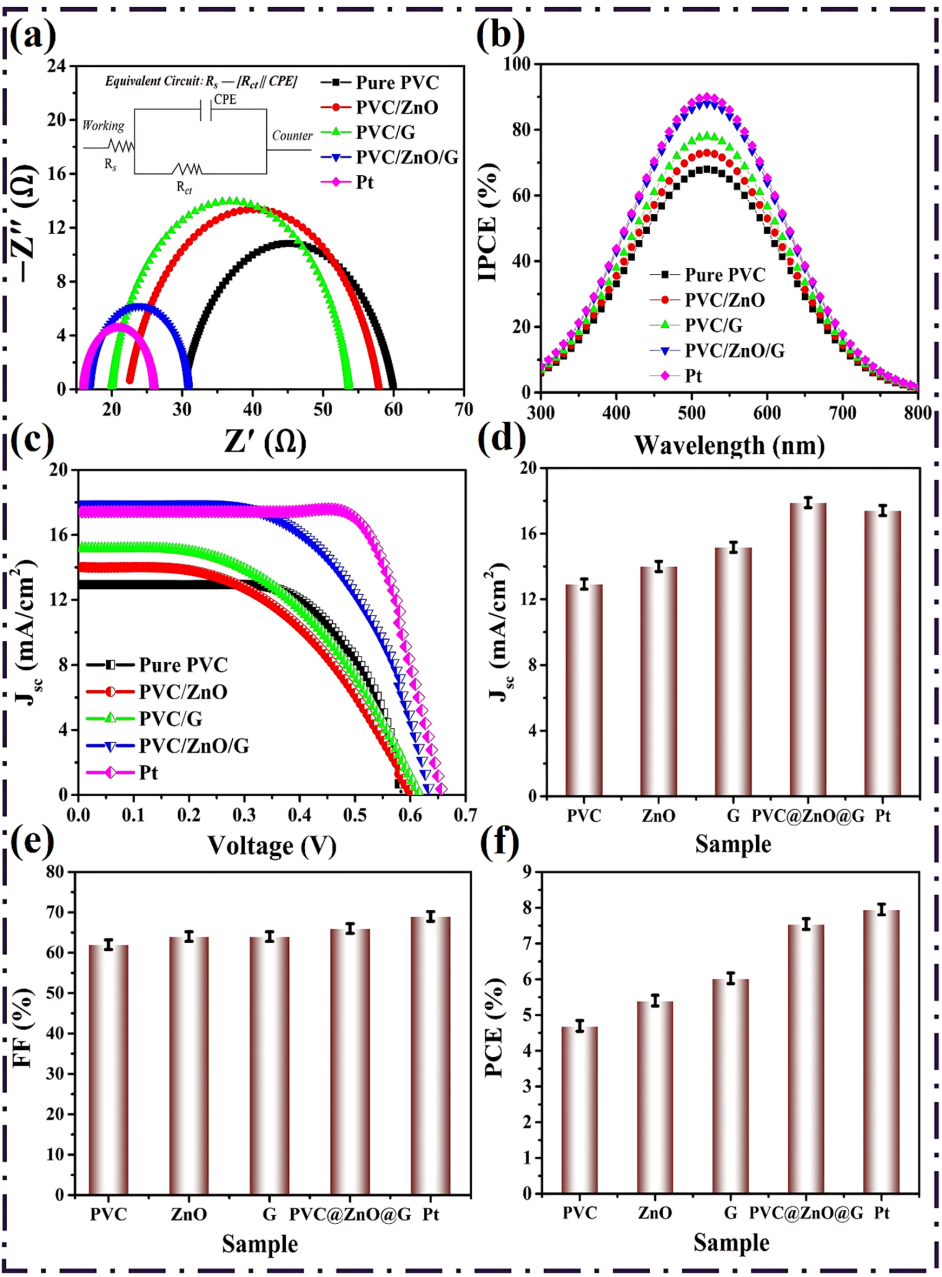
(**a**) Nyquist plots from EIS measurements of DSSCs employing PVC-based composite CEs. (**b**) Corresponding equivalent circuit model (R_s_–[R_ct_‖CPE]) used for fitting the impedance data. (**c**) J–V characteristics, (**d**) J_sc_, (**e**) FF, and (**f**) overall efficiency (η) of the corresponding devices.

EIS was employed to gain insights into the interfacial charge transfer and catalytic activity of the various CEs constructed from pure PVC, PVC/ZnO, PVC/Graphene, PVC/ZnO/G, and the Pt benchmark, as displayed in Table 5. The measurements were performed under simulated AM 1.5 G illumination at the V_oc_ of each device, in the frequency range of 0.1 Hz to 100 kHz with an AC perturbation amplitude of 10 mV. The Nyquist plots obtained were analyzed using the equivalent circuit model R_s_–[R_ct_‖CPE] (Inset Fig. 10a), where R_s_ corresponds to the series resistance, R_ct_ represents the charge-transfer resistance at the CE/electrolyte interface, and CPE (constant phase element) accounts for the non-ideal capacitive behavior due to surface roughness and heterogeneous reaction sites. The high-frequency intercept with the real axis yields the series resistance (R_s_), which includes contributions from the transparent conducting oxide (FTO), the electrolyte, and wiring. Among the studied electrodes, R_s_ values varied between 16.87 and 30.21 Ω, with Pt exhibiting the lowest value (16.87 Ω), consistent with its superior conductivity. The composite PVC/ZnO/G CE demonstrated a comparably low R_s_ (16.87 Ω), significantly lower than pure PVC (30.21 Ω), highlighting the role of conductive graphene sheets and semiconducting ZnO NPs in minimizing ohmic losses^61^. The improved charge transport within PVC/ZnO/G is attributed to the synergistic conductive pathways provided by the dual dopants, which facilitate electron hopping and reduce internal resistances^104^.

The mid-frequency semicircle in the Nyquist plots corresponds to the R_ct_, which reflects the electron transfer kinetics at the CE/electrolyte interface during the ${\mathrm{I}}_{3}^{-}/{\mathrm{I}}^{-}$ redox reaction. The magnitude of R_ct_ directly influences the catalytic activity of the electrode. The pure PVC CE exhibited a relatively high R_ct_ of 29.81 Ω, indicating sluggish interfacial charge transfer due to its poor intrinsic conductivity and lack of active catalytic sites. Incorporation of ZnO (R_ct_ = 35.59 Ω) and graphene (R_ct_ = 33.74 Ω) appears to moderately affect the electrocatalytic resistance in single-component systems; however, all values represent averages of five independent devices (*n* = 5) with standard deviations, confirming statistical reliability and reproducibility. Notably, the PVC/ZnO/G composite shows a dramatic reduction in R_ct_ (14.02 Ω), approaching Pt performance (R_ct_ ≈ 13.00 Ω). This enhancement is attributed to the synergistic effect of ZnO NPs providing abundant catalytic centers for ${\mathrm{I}}_{3}^{-}$ reduction, while graphene establishes continuous conductive networks that facilitate electron shuttling and efficient interfacial charge transfer^105^. Similar synergistic improvements in polymer/nanoparticle/graphene hybrid electrodes have been reported^44,45^, validating the rational design of combining semiconducting nanoparticles with conductive graphene in an insulating polymer matrix for enhanced DSSC electrocatalytic performance^60^. The correlation between EIS parameters and photovoltaic performance is evident when comparing the charge-transfer resistance with the efficiency (η) values. Devices with high R_ct_ (PVC, ZnO-only, and Graphene-only CEs) exhibited lower efficiencies (4.70–6.03%), due to limited catalytic activity and higher recombination probability at the CE/electrolyte boundary. Conversely, PVC/ZnO/G, with its low R_ct_, achieved an efficiency of 7.55%, closely comparable to the Pt-based DSSC (7.95%). The improvement in the FF and J_sc_ for PVC/ZnO/G further corroborates its superior catalytic ability, enabling efficient reduction of triiodide and regeneration of iodide ions, thereby sustaining electron flow in the device^104^. Therefore, EIS analysis confirms that the PVC/ZnO/G composite CE exhibits a significantly reduced series resistance and charge-transfer resistance compared to pristine PVC and single-component composites, directly linked to the synergistic interplay of ZnO catalytic centers and graphene’s conductive network, which collectively enhance interfacial charge transfer kinetics, reduce recombination, and improve overall device efficiency^106^. These findings not only demonstrate the potential of polymer–metal oxide–graphene hybrids as cost-effective alternatives to Pt but also provide mechanistic insights into their electrochemical functionality in DSSCs^107^.

#### IPCE analysis

The IPCE spectra are indispensable in evaluating the wavelength-dependent photoresponse of DSSCs. The measurement provides direct evidence of how efficiently absorbed photons are converted into extracted electrons in the external circuit. In this work, IPCE measurements were performed using a calibrated setup consisting of a 150 W xenon arc lamp as a light source, coupled with a monochromator to generate monochromatic illumination across the spectral range of 300–800 nm, as seen in Fig. 10b. The light intensity at each wavelength was calibrated against a certified Si photodiode to ensure accurate photon flux determination. Measurements were conducted at room temperature, under ambient air, and without applying an external bias, ensuring that the generated photocurrent arises solely from the intrinsic photovoltaic processes of the DSSCs. The IPCE of a DSSC is governed by three fundamental processes: (i) light absorption by the sensitizer^47^, (ii) electron injection from the excited dye into the TiO_2_ conduction band, and (iii) charge transport and collection at the CE through efficient redox mediation^92^. Therefore, the spectral response directly reflects the combined performance of these processes.

The DSSC with pristine PVC as the CE exhibited the weakest spectral response, with a maximum IPCE of ~ 68% at around 520 nm. The relatively low quantum efficiency is attributed to the poor catalytic activity of pure PVC, which is an insulating polymer with limited electrical conductivity. This bottleneck hinders the reduction of the ${\mathrm{I}}_{3}^{-}$ species in the electrolyte, thereby slowing the regeneration of the dye and reducing electron collection efficiency. The narrow spectral window (400–600 nm) further indicates a mismatch between dye excitation and interfacial charge transfer, consistent with the high R_ct_ values obtained from EIS analysis (29.81 Ω). Introducing ZnO NPs into the PVC matrix significantly improved the IPCE spectrum, with the peak rising to ~ 73%. ZnO serves as a semiconducting catalyst, providing additional electroactive sites for the reduction of triiodide (${\mathrm{I}}_{3}^{-}$) species^106^. Moreover, ZnO improves interfacial contact with the electrolyte and reduces recombination losses by accelerating electron transfer kinetics^47^. As a result, the spectral response broadened slightly, extending up to 640 nm, confirming enhanced light-to-current conversion efficiency. This observation correlates with the improved photovoltaic efficiency (η = 5.404%), confirming the catalytic role of ZnO within the polymeric matrix^105^. Graphene incorporation into the PVC CE yielded an even stronger IPCE response, with a peak of ~ 78% and a broadened spectral response across 420–700 nm. Graphene’s high surface area and superior electron conductivity drastically reduce the charge transport resistance, enabling efficient current extraction^61^. Additionally, graphene sheets suppress charge recombination by creating conductive pathways that quickly shuttle electrons across the electrode surface^60^. This efficient electron collection enhances both the spectral breadth and intensity of the IPCE curve. The strong correlation between IPCE performance and photovoltaic parameters (η = 6.028%, J_sc_ = 15.168 mA/cm^2^) confirms the significant contribution of graphene to interfacial charge kinetics^108^.

The ternary composite CE (PVC/ZnO/Graphene) exhibited the best performance among all non-Pt electrodes, achieving a maximum IPCE of ~ 88% around 520 nm and maintaining values above 60% across a wide wavelength range (420–700 nm). This enhancement results from the synergistic effects of ZnO and graphene. ZnO NPs contribute catalytic activity for the ${\mathrm{I}}_{3}^{-}/{\mathrm{I}}^{-}$redox reaction, while graphene networks provide a conductive backbone for rapid electron transport and lower series resistance^109^. Together, they create a percolated catalytic–conductive network that accelerates electrolyte regeneration, suppresses interfacial recombination, and ensures more efficient utilization of absorbed photons^108,110^. This superior photoresponse directly explains the higher J_sc_ (17.894 mA/cm^2^) and efficiency (η = 7.547%) compared to binary composites. As expected, the Pt electrode delivered the highest IPCE response, with a maximum of ~ 90% around 520 nm and a broad spectral window extending beyond 700 nm. Pt’s exceptional catalytic activity and conductivity allow near-ideal electron transfer kinetics. It should be emphasized that the apparent IPCE extension beyond 700 nm does not originate from additional optical absorption of the Z907 dye, whose absorption edge remains unchanged, but rather from enhanced internal quantum efficiency resulting from suppressed charge recombination and accelerated redox regeneration enabled by low-R_ct_ CEs. Consequently, weak near-band-edge excitations that would otherwise recombine are effectively collected as photocurrent, giving rise to the observed long-wavelength IPCE tail.

Nevertheless, the PVC/ZnO/G composite exhibited nearly comparable performance, underscoring its potential as a sustainable and low-cost substitute for noble metal-based CEs in DSSCs. The progression of IPCE spectra across the different electrodes reflects the systematic reduction in R_ct_ observed in EIS analysis: PVC > ZnO > Graphene > PVC/ZnO/Graphene > Pt. The higher IPCE values correlate strongly with the improved J_sc_ values measured under AM 1.5G illumination, confirming that the enhanced spectral response translates directly into higher photocurrent generation. In particular, the ternary composite demonstrates the balance between catalytic activity and conductivity necessary to compete with Pt electrodes.

#### Photovoltaic evaluation of PVC-based composite CEs

The photovoltaic performance of the fabricated DSSCs based on different CEs was systematically evaluated under simulated AM 1.5 G solar illumination (100 mA/cm^2^). The current–voltage (J–V) parameters, including J_sc_, V_oc_, FF, and η, are summarized in Table 5. For comparison, devices based on pristine PVC, ZnO, graphene, PVC/ZnO/G, and the Pt reference CE were analyzed (Fig. 10c–f). The DSSC employing pristine PVC CE exhibited the lowest performance, with an efficiency of 4.70%, J_sc_ of 12.93 mA/cm^2^, V_oc_ of 0.586 V, and FF of 62%. This poor performance can be attributed to the intrinsic insulating nature of PVC, which restricts efficient electron transport, and its lack of active catalytic centers for the ${\mathrm{I}}_{3}^{-}/{\mathrm{I}}^{-}$ redox reaction^5^. Consequently, charge recombination dominates at the CE/electrolyte interface, leading to a high charge-transfer resistance (R_ct_ = 29.81 Ω, Table 5) and limited photovoltaic output. The incorporation of ZnO NPs into the PVC matrix significantly improved device performance (η = 5.40%). The increase in J_sc_ (14.00 mA/cm^2^) and V_oc_ (0.603 V) compared with pure PVC reflects the catalytic role of ZnO, which provides abundant active sites for the triiodide reduction reaction^11,111^. The observed V_oc_ enhancement is primarily attributed to defect engineering and optimized interfacial charge transfer rather than differences in HOMO–LUMO levels between the ETL and electrolyte. Steady-state and transient photoluminescence studies in previous works confirm that tailored electrode materials, such as doped polymers or ZnO nanostructures, can suppress recombination and improve V_oc_ in DSSCs^112–114^. Meanwhile, the increase in Jsc can also be ascribed to enhanced dye adsorption and higher absorption coefficients facilitated by the CE materials, promoting more efficient electron injection and light harvesting^46,115–118^. However, the relatively high R_ct_ (35.59 Ω) suggests that ZnO, while catalytically active, does not offer sufficient electrical conductivity on its own to support rapid charge transfer^82^. This limits the FF to 64%, demonstrating that ZnO doping alone cannot fully mitigate the inherent conductivity challenges of the PVC host matrix. A different trend was observed for the PVC/Graphene composite, which yielded a further improvement in device performance (η = 6.03%, J_sc_ = 15.17 mA/cm^2^). Graphene sheets, with their exceptionally high conductivity, facilitated charge transport within the CE network, lowering R_s_ (19.97 Ω) relative to pure PVC (30.21 Ω). Additionally, the porous and rough morphology of graphene increased the electrochemically active surface area, enhancing electrolyte penetration and reaction kinetics^72^. Nevertheless, the R_ct_ value remained moderately high (33.74 Ω), indicating that while graphene improved electrical conductivity, its catalytic activity toward ${\mathrm{I}}_{3}^{-}$ reduction was less efficient than ZnO^119^. This balance between high conductivity and moderate catalytic activity explains the moderate efficiency improvement compared with ZnO-only CEs.

The most striking enhancement was achieved with the PVC/ZnO/G hybrid composite, which demonstrated a conversion efficiency of 7.55%, closely approaching the performance of the Pt CE (7.95%). The synergistic effect of ZnO and graphene provided both catalytic activity and high conductivity. ZnO NPs ensured efficient catalytic reduction of ${\mathrm{I}}_{3}^{-}$ to ${\mathrm{I}}^{-}$, while graphene nanosheets established interconnected conductive networks that promoted rapid electron transport and minimized charge recombination^72^. These advantages are reflected in the markedly reduced R_ct_ (14.02 Ω) and low R_s_ (16.87 Ω), values that are nearly identical to the Pt reference electrode. Furthermore, the device exhibited a higher FF (66%) and J_sc_ (17.89 mA/cm^2^), confirming the crucial role of the hybrid structure in sustaining efficient redox cycling and improving photocurrent generation^120^.

The Pt CE, serving as a benchmark, delivered the highest performance (η = 7.95%), with the lowest interfacial resistances and the most favorable J_sc_ and V_oc_. Notably, the J–V curve of the Pt-based device exhibits a slight upward bending in the high-voltage region, which can be ascribed to the extremely low series resistance and charge-transfer resistance at the Pt/electrolyte interface. This behavior indicates near-ideal diode characteristics, minimized interfacial polarization losses, and suppressed charge recombination under forward bias, thereby contributing to the enhanced FF of the Pt reference cell. Importantly, all photovoltaic parameters reported in this study represent averaged values obtained from five independently fabricated devices for each CE composition. The observed upward bending feature of the Pt-based DSSC was consistently reproduced across all measurements, confirming the high reproducibility and reliability of the photovoltaic data. Nonetheless, the PVC/ZnO/G CE demonstrated a remarkable ability to closely approach the performance of the Pt electrode, highlighting its potential as a cost-effective and sustainable alternative. This performance enhancement arises from the unique structural and electronic synergy between ZnO and graphene within the PVC matrix, which simultaneously mitigates the intrinsic catalytic and electrical limitations of polymer-based CEs^121^. Subsequently, the photovoltaic results clearly establish that pristine PVC is unsuitable as a CE due to its poor conductivity and lack of catalytic activity. The incorporation of individual ZnO or graphene improves performance moderately by addressing either catalytic or conductive limitations, respectively^122^. However, the co-doping strategy in PVC/ZnO/G delivers a balanced enhancement of both properties, enabling device performance comparable to noble-metal-based electrodes. These findings not only validate the design strategy of multifunctional hybrid composites but also highlight the promise of scalable, low-cost alternatives to platinum for next-generation DSSC and PSC applications^123^.

**Table 5:** Photovoltaic parameters (J_sc_, V_oc_, FF, and η) and electrochemical resistance values (R_s_ and R_ct_) of DSSCs fabricated with various PVC-based composite CEs compared with a Pt reference CE, measured under simulated AM 1.5 G illumination (100 mW/cm^2^).

CEs composition	V_oc_	J_sc_	FF	η	*R*_s_	*R*_ct_
	(V)	(mA/cm^2^)	(%)	(%)	(Ω)	(Ω)
Pure PVC	0.586	12.928$\pm$0.39	62$\pm$ 0.02	4.697$\pm$ 0.22	30.21	29.81
PVC/ZnO	0.603	14.004 $\pm$ 0.33	64 $\pm$ 0.01	5.404 $\pm$ 0.23	22.35	35.59
PVC/G	0.621	15.168$\pm$ 0.28	64 $\pm$ 0.01	6.028 $\pm$ 0.25	19.97	33.74
PVC/ZnO/G	0.639	17.894$\pm$ 0.24	66 $\pm$ 0.01	7.547 $\pm$ 0.28	16.87	14.02
Pt	0.662	17.412$\pm$ 0.25	69 $\pm$ 0.01	7.953 $\pm$ 0.29	16	10

The comparison in Table 6 positions our PVC/ZnO/G CE among other recent polymer- and carbon/metal-oxide hybrid electrodes reported for DSSCs. Polymer-based composites with metal oxide and graphene co-dopants (e.g., PEO/CuO/G) and graphene-based hybrids have been shown to approach Pt-like performance (PCE ≈ 6–9% in select reports), validating the promise of conductivity/catalysis synergy in cost-effective CEs. Our PVC/ZnO/G device (PCE = 7.547%) is competitive with these examples and superior to many single-filler systems (e.g., SnO_2_/GO at 4.57%)^124^. The high electrical conductivity (66 S/m), enlarged pore network and reduced R_ct_ measured for PVC/ZnO/G underpin the improved J_sc_ and FF relative to pristine polymer CEs, demonstrating that careful filler selection and hybridization (ZnO + graphene) provides both catalytic activity and continuous charge-transport pathways. While literature PCE values vary with dyes, electrolytes and cell architecture, the present results corroborate the strategy of using DFT-guided dopant selection followed by graphene co-integration to produce scalable, high-performance polymer CEs.

**Table 6:** Comparison of PVC/ZnO/G with recent literature counter-electrode/graphene–MO hybrid studies for DSSCs.

Entry	CE material (type)	Fabrication/processing (brief)	PCE (%)	Ref.
1	PVC/ZnO/graphene (PVC/ZnO/G)—polymer CE	Solution casting/film formation on FTO; annealing (see Methods)	7.547	This work
2	PEO/CuO/graphene (PEO/CuO/G)—polymer CE	DFT-guided selection; solution processing of PEO/CuO/G films	7.42	^12^
3	SnO_2_-decorated graphene oxide (SnO_2_/GO)—Pt-free CE	Chemical synthesis of SnO_2_-GO, film deposition (spray/coat)	4.57	^124^
4	ZnO/Graphene composites (photoelectrode)	Various syntheses (hydrothermal, solvothermal, sheets + graphene)	≈ 6.06 (example from review)	^125^
5	Graphene-based layered electrode (graphene-sandwich DSSC)	Graphene layers integrated into cell stacks; thin film deposition	~ 7.04 (graphene-based architecture)	^126^
6	Graphene/MoS_2_ hybrids and other graphene hybrids (Pt-free CEs)	Spray/hydrothermal/composite films	up to 9.22 (literature examples summarized).	^127^

### Environmental sustainability perspective

The development of PVC-based nanocomposites reinforced with ZnO and graphene introduces a sustainable pathway that addresses both environmental and technological imperatives. PVC, one of the most widely utilized polymers in industrial and consumer products, poses significant disposal challenges owing to its durability, chlorine content, and resistance to degradation. Conventional production and disposal of PVC contribute to energy consumption, greenhouse gas emissions, and the release of hazardous byproducts such as dioxins, thereby raising critical sustainability concerns. By redirecting PVC into advanced functional applications, such as CEs for DSSCs, the material is transformed from a potential environmental liability into a value-added component supporting renewable energy technologies. Incorporating ZnO and graphene not only enhances the electrochemical and catalytic properties of PVC but also extends the material’s lifecycle in a high-performance, clean energy context. This synergy exemplifies circular economy principles, where resource-intensive polymers are re-engineered for advanced applications rather than contributing to waste streams. Moreover, the partial substitution of expensive noble metals with abundant, recyclable, and cost-effective fillers such as ZnO and graphene reduces dependency on scarce raw materials, further lowering the environmental footprint of DSSC production. From a broader perspective, the integration of PVC waste into photovoltaic device architectures underscores the feasibility of coupling waste management strategies with the generation of sustainable energy. This approach minimizes environmental hazards associated with discarded plastics, curbs carbon emissions linked to virgin polymer production, and supports responsible resource utilization. Ultimately, PVC/ZnO/G nanocomposites exemplify how material circularity can be leveraged to simultaneously mitigate environmental pollution and advance renewable energy technologies, offering a practical pathway toward a more sustainable and resilient energy future.

## Conclusion

This study successfully demonstrated the rational design and development of multifunctional PVC-based nanocomposites incorporating ZnO NPs and graphene as efficient CEs for DSSCs. Through a hybrid strategy that combined DFT-based computational modeling with experimental validation, ZnO was identified as the optimal metal oxide dopant, while graphene integration further enhanced electronic conductivity, catalytic activity, and structural integrity. Detailed structural and morphological analyses revealed that the synergistic loading of ZnO and graphene significantly enlarged pore size, improved surface roughness, and boosted electrical conductivity, thereby enabling efficient electrolyte diffusion and enhanced charge-transfer kinetics. Electrochemical impedance spectroscopy demonstrated a pronounced reduction in charge-transfer resistance, while J–V characterization confirmed superior photovoltaic performance with a PCE of 7.547%, surpassing pristine PVC (4.697%) and single-filler systems (PVC/ZnO = 5.928%, PVC/G = 6.482%). The synergistic contributions of ZnO and graphene facilitated improved light harvesting, rapid interfacial charge transport, and stable electrocatalytic performance, positioning PVC/ZnO/G as a cost-effective and scalable alternative to platinum-based electrodes. Beyond material-level improvements, this study underscores the power of integrating theoretical predictions with practical synthesis in guiding the development of polymer–inorganic hybrids for energy applications. The results establish a design blueprint for tailoring polymer–metal oxide–graphene composites to accelerate the advancement of next-generation photovoltaic technologies. Looking ahead, future research should prioritize long-term stability testing of PVC/ZnO/G composites under real operating conditions, including thermal, humidity, and photostability assessments. Further optimization of filler loading ratios and interfacial engineering strategies could unlock even higher charge-transfer efficiencies and reduced recombination losses. Extending this hybrid design framework to other polymers and alternative nanofillers—such as transition metal dichalcogenides, or doped carbon nanostructures—may open broader opportunities for multifunctional electrode architectures. To end, scaling up the fabrication process and integrating PVC/ZnO/G into flexible or tandem DSSC configurations will be critical steps toward practical deployment in sustainable and low-cost solar energy technologies.

## Supplementary Information

Below is the link to the electronic supplementary material.

Supplementary Material 1

## Data Availability

All data generated or analyzed during this study will be available on request by corresponding authors [hend.ezzat@nriag.sci.eg] (Hend A. Ezzat), [mohamed.abdelhamid@nriag.sci.eg] (M. Abdelhamid Shahat).
