# Supplementary material for: Multifunctional PVC-based metal oxide/graphene composites for high-performance DSSC counter electrodes
Source: Sci Rep. 2026 Mar 25;16:9817. doi: 10.1038/s41598-026-41857-w (PMC13018560; doi:10.1038/s41598-026-41857-w)
Supplement: Supplementary file 1 — Supplementary Material 1 [file 41598_2026_41857_MOESM1_ESM.docx]

**Supplementary Information – EIS Analysis**

Full-scale Nyquist plots with original axis scaling are provided in the Supplementary Information (Fig. S1) to demonstrate the complete EIS response of all counter electrodes. The extracted electrochemical parameters remain unchanged and consistent with those discussed in the main text.


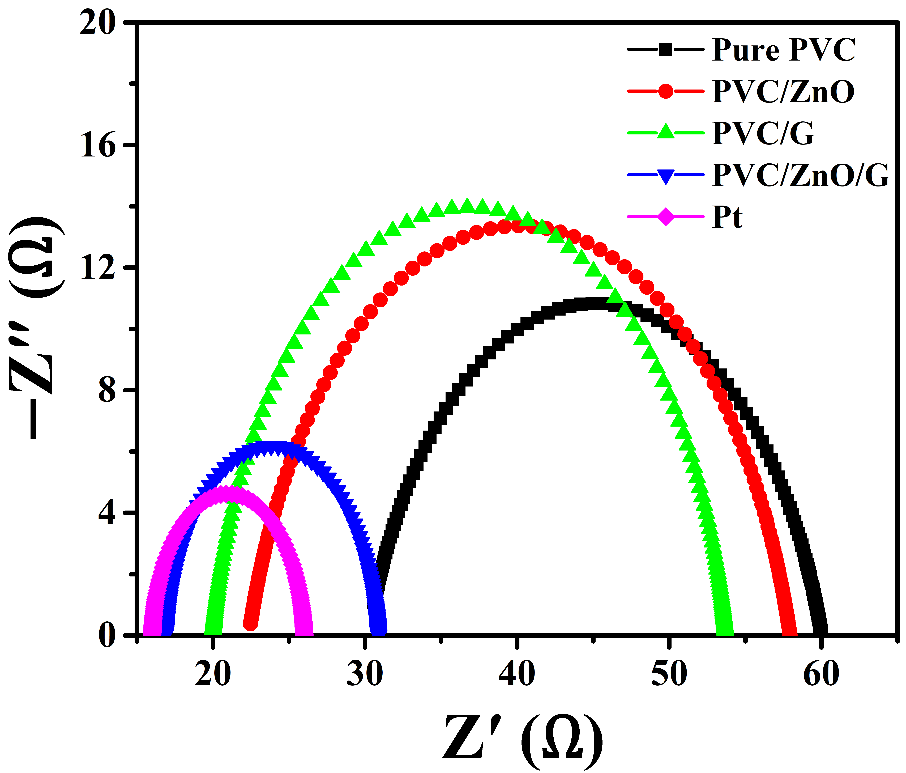


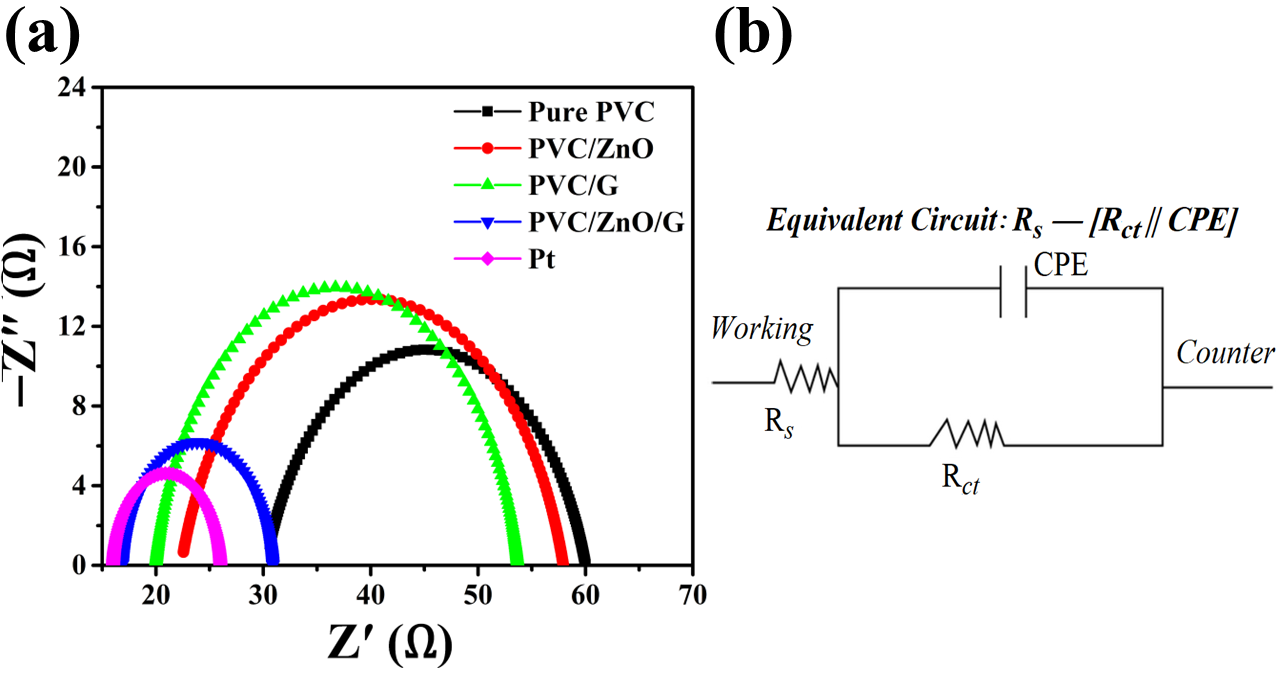


**Figure S1.** Full-scale Nyquist plots of DSSCs fabricated with pure PVC, PVC/ZnO, PVC/Graphene, PVC/ZnO/Graphene, and Pt counter electrodes, measured over the frequency range of 0.1 Hz–100 kHz under AM 1.5G illumination. The spectra are presented using the original, unadjusted x- and y-axis scales to display the complete electrochemical response of each device. The extracted R_s_ and R_ct_ values are consistent with those obtained from the scaled Nyquist plots shown in Fig. 10a and summarized in Table 5, confirming the reliability and accuracy of the EIS fitting and analysis.
